# Multi-modal data collection for measuring health, behavior, and living environment of large-scale participant cohorts

**DOI:** 10.1093/gigascience/giab044

**Published:** 2021-06-21

**Authors:** Congyu Wu, Hagen Fritz, Sepehr Bastami, Juan P Maestre, Edison Thomaz, Christine Julien, Darla M Castelli, Kaya de Barbaro, Sarah Kate Bearman, Gabriella M Harari, R Cameron Craddock, Kerry A Kinney, Samuel D Gosling, David M Schnyer, Zoltan Nagy

**Affiliations:** Department of Psychology, University of Texas at Austin, 108 E Dean Keeton St, Austin, Texas, 78712, USA; Department of Civil, Environmental, and Architectural Engineering, University of Texas at Austin, 301 E Dean Keeton St, Austin, Texas, 78712, USA; Department of Civil, Environmental, and Architectural Engineering, University of Texas at Austin, 301 E Dean Keeton St, Austin, Texas, 78712, USA; Department of Civil, Environmental, and Architectural Engineering, University of Texas at Austin, 301 E Dean Keeton St, Austin, Texas, 78712, USA; Department of Electrical and Computer Engineering, University of Texas at Austin, 2501 Speedway, Austin, Texas, 78712, USA; Department of Electrical and Computer Engineering, University of Texas at Austin, 2501 Speedway, Austin, Texas, 78712, USA; Department of Kinesiology and Health Education, University of Texas at Austin, 2109 San Jacinto Blvd, Austin, Texas, 78712, USA; Department of Psychology, University of Texas at Austin, 108 E Dean Keeton St, Austin, Texas, 78712, USA; Department of Educational Psychology, University of Texas at Austin, 1912 Speedway, Austin, Texas, 78712, USA; Department of Communication, Stanford University, 450 Serra Mall, Stanford, California, 94305, USA; Department of Diagnostic Medicine, University of Texas at Austin, 1601 Trinity St, Austin, Texas, 78712, USA; Department of Civil, Environmental, and Architectural Engineering, University of Texas at Austin, 301 E Dean Keeton St, Austin, Texas, 78712, USA; Department of Psychology, University of Texas at Austin, 108 E Dean Keeton St, Austin, Texas, 78712, USA; Melbourne School of Psychological Sciences, University of Melbourne, Grattan Street, Parkville, Victoria, 3010, Australia; Department of Psychology, University of Texas at Austin, 108 E Dean Keeton St, Austin, Texas, 78712, USA; Department of Civil, Environmental, and Architectural Engineering, University of Texas at Austin, 301 E Dean Keeton St, Austin, Texas, 78712, USA

**Keywords:** multi-modal sensing, human-centered computing, smartphone, Fitbit, BEVO Beacon, ecological momentary assessment, health, college students

## Abstract

**Background:**

As mobile technologies become ever more sensor-rich, portable, and ubiquitous, data captured by smart devices are lending rich insights into users’ daily lives with unprecedented comprehensiveness and ecological validity. A number of human-subject studies have been conducted to examine the use of mobile sensing to uncover individual behavioral patterns and health outcomes, yet minimal attention has been placed on measuring living environments together with other human-centered sensing data. Moreover, the participant sample size in most existing studies falls well below a few hundred, leaving questions open about the reliability of findings on the relations between mobile sensing signals and human outcomes.

**Results:**

To address these limitations, we developed a home environment sensor kit for continuous indoor air quality tracking and deployed it in conjunction with smartphones, Fitbits, and ecological momentary assessments in a cohort study of up to 1,584 college student participants per data type for 3 weeks. We propose a conceptual framework that systematically organizes human-centric data modalities by their temporal coverage and spatial freedom. Then we report our study procedure, technologies and methods deployed, and descriptive statistics of the collected data that reflect the participants’ mood, sleep, behavior, and living environment.

**Conclusions:**

We were able to collect from a large participant cohort satisfactorily complete multi-modal sensing and survey data in terms of both data continuity and participant adherence. Our novel data and conceptual development provide important guidance for data collection and hypothesis generation in future human-centered sensing studies.

## Introduction

Human health and behavioral research is primarily conducted in laboratories under conditions that poorly approximate real-world conditions. While this model has been successful, it may miss key aspects of human behaviors that are elicited only during more natural conditions or interactions. This concern has driven interest in developing and using remote sensing technologies to measure individuals completing their normal day-to-day activities in their natural environment. An explosion in modern technologies, many of which are in common use, now provide the ability to monitor and understand health and human behavior in ways not previously possible. Smartphones, smart home devices, wearables, and online digital behaviors provide new ways to track sleep, emotions, spatial mobility, activity, environmental exposures, and social interactions to name just a few. These technological advances offer opportunities to unobtrusively collect real-time data on a wide range of social-behavioral and health variables with less participant burden and more ecological validity than ever before [1].

In the past decade we have seen growing effort worldwide in collecting real-time sensing and experience-sampling data from human participants in natural, uncontrolled settings [2–6]. Smartphones are the staple of sensing hardware to measure different aspects of daily behavior [7]. Four main categories of behavioral patterns can be captured passively: (i) mobility trajectories, measured by GPS and further processed into location clusters that represent significant places visited [8]; (ii) physical activity, measured by accelerometer and further processed into activity status labels such as walking and staying still [9]; (iii) social context, reflected in different modes including telephone calls, message exchange, and physical proximity detected by Bluetooth, which can be used to reconstruct social networks [10]; and (iv) interaction with the device, such as screen unlock status and app use, which are logged by the smartphone itself [11]. Additionally, ecological momentary assessment (EMA) surveys can be deployed to actively collect participants' self-reports of mood, behavior, and well-being in real time.

While many aspects of behavior and health can be captured in smartphone sensing data including EMAs with satisfactory ecological validity, we realize that other key dimensions of well-being are better measured using complementary technologies. A person spends a substantial proportion of their time at home, but measurements of their home environment are not generally investigated in existing studies in parallel with other aspects of daily behavior. To this end, we developed a home environment sensing device, named “BEVO Beacon," that is capable of continuously collecting and uploading multiple measures of indoor air quality. This device can provide critical insights into a participant’s living environment and evaluate its behavioral and health implications. Additionally, sleep is a critical health outcome and independent variable that is difficult to measure objectively using unobtrusive instruments. We argue that it is especially beneficial to utilize the sleep-measuring capability of wearable devices such as Fitbit to validate EMA answers.

Furthermore, the vast majority of existing human sensing studies used fewer than a few hundred participants [12]. A larger sample is needed to obtain more reliable assessments of the correlations between key behavior and health measures, especially when a large number of variables is assessed. Reflecting these considerations, we conducted a multi-modal human sensing study named UT1000, for which we recruited >1,000 college students as participants over 2 deployments and distributed a variety of sensors and instruments including smartphone, Fitbit, BEVO Beacon, and EMA. The resulting data allow us to pursue research questions that previous data were unable to accommodate.

With numerous types of technologies and methods potentially available to measure individual humans’ health, behavior, and environment, we begin in the next Section by proposing a novel conceptual framework that organizes the various modalities of human-centric data based on their properties. Then, we present the study design and the types of data collected in our UT1000 Project in Section “The UT1000 Project". We visualize the concurrent data streams collected during the study and evaluate their completeness in Section “Data Validation". In Section “Reuse Potential" we outline potential analytical and research applications for which our data can be useful. Through this study we are able to gain comprehensive understandings of the lives of college students and learn valuable lessons about the design, deployment, and data analysis for large-scale human sensing studies.

## Conceptual Framework

**Figure 1:**
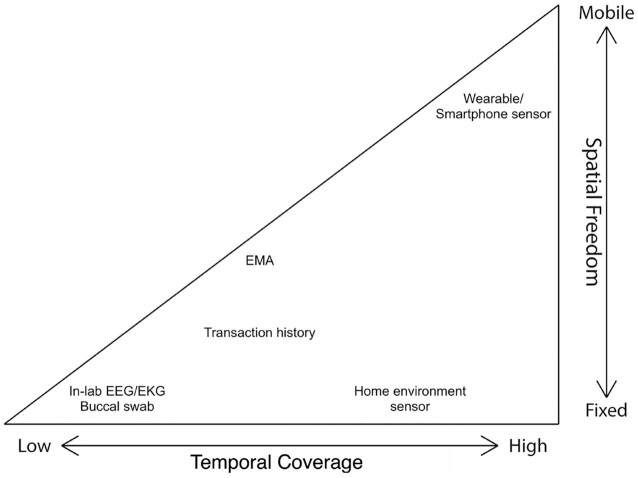
Human-centric data modality framework.

Figure 1 illustrates the conceptual framework that we devised for organizing different technologies and methods for observing human outcomes based on properties of their data collection procedure and resulting data.

A primary property of human-centric data is its **temporal coverage**, represented by the x-axis from low (left) to high (right). Temporal coverage is defined by the inherent suitability of a data modality to monitor extended proportions of time of an individual’s daily life. Data modalities that can provide only single-time observations are on the low end of the temporal coverage dimension. Examples include (i) traditional survey inventories that are designed to provide a 1-time diagnosis of a potential patient and (ii) medical procedures that typically require in-person clinical visits such as electroencephalography, electrocardiography, and buccal swab sampling. Some data collection methods or technologies accommodate measurements taken at multiple points in time, thus are placed mid-range along the temporal coverage dimension. Examples include (i) self-reports in response to EMAs delivered via mobile devices and (ii) record data such as transaction history, which contains user information logged at different times of user engagement with a service or services. The highest temporal coverage is achieved by continuous tracking. Various sensors embedded in devices that people carry where they go and install where they stay belong in this category, such as smartphones, wearable devices, and environmental sensors used as smart home technology. Wearable devices including smartphones tend to benefit from an even higher level of temporal coverage than environmental sensors. This is because wearable devices are able to accompany and monitor the user at all times as long as the user keeps the devices on their person and powered on, thus observing the user for longer periods of time compared to environmental sensors, which can only offer human-centric sensing capability when the individual in question remains within their proximity (e.g., home environment sensors are only human-centric when the person is at home).

A second property of human-centric data is their **spatial freedom**, represented by the y-axis in Fig. 1 ranging from fixed (bottom) to mobile (top). Spatial freedom is defined by the ability of a data modality to reflect an individual’s health, behavior, and environment at a variety of locations. Data modalities with greater temporal coverage tend to permit a wider range of locations where an individual can be monitored, a.k.a. higher spatial freedom. For this reason the coordinate space presented in Fig. 1 is a triangle. A discrete-time measurement of low temporal coverage is typically taken at a specific location, only allowing minimal spatial freedom, thus occupying the left vertex of the triangle. However, spatial freedom may vary greatly among instruments that can track users continuously over time. For example, both a PM2.5 sensor installed at home and a smart wristband can be considered as having high temporal coverage; however, they correspond to fixed and mobile, respectively, on the spatial freedom dimension.

The temporal coverage and spatial freedom of a data modality is often governed by the unobtrusiveness [13] of the technology or method producing it. Data modalities of higher temporal coverage and spatial freedom are usually produced by devices and procedures that are more user-friendly, more portable, and overall less burdensome for the user. Unobtrusive methods allow for more naturalistic and non-interfering ways to monitor a participant’s daily life, thus producing measures of greater ecological validity. We identify a major correlation between ecological validity and temporal coverage/spatial freedom: measures of high ecological validity tend to enable observations over extended periods of time and with greater mobility. However, the relation between ecological validity and temporal coverage/spatial freedom is not a necessary one. In the classic example of the study by Barker and Wright of Raymond Birch [14], in which a research team followed an individual around for a whole day making observations every few minutes, temporal coverage and spatial freedom are both high; however, ecological validity is low because the observation method was extremely intrusive.

The example data modalities shown in Fig. 1 are based on how the generating methods are naturally and realistically carried out. For example, a buccal swab procedure takes a few minutes to complete but it requires a high level of participation and effort from the patient; therefore even though technically buccal swabs can be frequently administered, we still consider it to be a highly intrusive, 1-time measurement. Many human-monitoring technologies, over their course of development, have seen themselves ascend on the temporal coverage scale and often on spatial freedom as well. For example, blood glucose testing used to require clinical visits, thus the burden was high; however, as the technology for on-body continuous glucose monitoring becomes perfected, blood glucose measurements can be obtained with unprecedentedly high unobtrusiveness, allowing its temporal coverage and spatial freedom to increase as well.

A critical challenge in interdisciplinary research joining social sciences and engineering is the high-fidelity mapping between human-centric constructs and technology-advanced methods. Our framework provides an interface between the constructs and the methods based on their temporal and spatial characteristics and implications for ecological validity and unobtrusiveness. Thus it can help social scientists locate fitting methods to measure the constructs of interest. Furthermore, this framework can be used to guide data collection and hypothesis generation concerning interrelations of different aspects of human behavior. Researchers can delineate a subarea in the triangle to serve as the scope of their own data collection. Hypotheses or research questions can be straightforwardly formulated by linking 2 spots on the triangle and querying the relationship between the 2 corresponding data modalities. Moreover, the 2D space describes 1 individual and can be conceptually stacked up to represent a group of individuals and their data and descriptors. Linkage between slices representing different individuals can inform the generation of research questions into the relations between individual outcomes and interpersonal interactions.

## The UT1000 Project

The UT1000 Project is a multi-modal data collection study conducted at the University of Texas at Austin to measure aspects of the health, behavior, and home environment of a large-scale participant cohort using a wide variety of technologies and methods, including traditional surveys, swabbing, EMAs, smartphone sensing, wearable trackers, and environmental sensors. We undertook 2 deployments, one in the fall of 2018 and the other in the spring of 2019, totaling 1,584 participants (62% female) and lasting 3 weeks each. Participants for this study were recruited through an introductory psychology course. Enrolled students were instructed to sign up for the EMA and smartphone sensing components of the study as a class assignment that counted toward their final grade. Students who did not want to self-track using a smartphone were given the option to record their behaviors and moods by answering e-mailed EMA questions or keeping a daily diary. The other data modalities such as those from the wearable trackers and environmental sensors, on the other hand, were collected in return for experimental credits, which the students used as partial fulfillment of the course requirements. The following subsections outline the different components of the UT1000 Project including the purpose, procedure, and types of data collected. Discussions of different study components are organized into 3 main categories based on the data modalities, namely, single-time, multiple-time, and continuous measures, following an order of temporal coverage from low to high consistent with the horizontal dimension of Fig. 1.

### Single-time measures

#### Home Environment and Health survey

The Home Environment and Health (HEH) questionnaire consists of 63 questions asking students to report on home environment factors such as their current living situation including number of roommates, number and type of pets, and flooring type; their recent health and medical histories including colds, allergies, and influenza shots received; and other behaviors such as hand-washing frequency and use of electric scooters. A full list of the HEH questions is provided in Additional File 1. The purpose of this survey was to obtain a better understanding of the participants’ home environment and to clarify discrepancies found in the other data streams.

The HEH questionnaire was a voluntary survey sent directly to participants via the e-mail address they provided to register for the study. Completing the HEH survey was a prerequisite for the subsequent home environment sensing component of the study. The survey was sent once during the first 2 weeks of the study period. Participants were asked to fill out the survey on the basis of their situation when they received it rather than some time in the past or the future. A total of 56 participants completed the HEH questionnaire, with 46 in fall 2018 and 10 in spring 2019.

#### Student environment and buccal swabbing

A subset of the study participants were provided with a dust sampling kit to collect dust samples from various surfaces in their home and classroom environment. The same participants who completed the HEH survey were given the kits (*N* = 56). The kit consisted of 6 individually packaged phosphate-buffered saline Tween-20 (PBST) wetted FLOQswabs^®^ (manufactured by COPAN, Murrieta, CA, USA) and 6 corresponding plastic resealable test tubes in which participants would place the swabs after collecting samples. Participants were asked for identification in order to gather sampling materials from a refrigerator-equipped storefront created ad hoc in a convenient place at a central location of the university. Testing materials to be distributed to different participants were labeled with distinct barcodes so that we could easily trace the materials back to the participants and streamline the checkout process.

Participants followed instructions to collect samples from the interior and exterior of their front door trim; cellphone screen; living room floor; heating, ventilation, and air conditioning (HVAC) filter or air diffuser if applicable; and a desktop where they normally sit when attending university classes. After sample collection, participants sealed swabs in the provided test tubes and placed them in their refrigerator until transportation to the university laboratory. When participants returned the testing materials, material barcodes were scanned, the identity of the participant cross-referenced to the materials, and the temperature-sensitive samples were stored until transfer to a −20°C freezer daily after storefront closure. They were asked to provide feedback on the challenges while performing home sampling and also whether they were willing to submit a buccal swab. If they consented, the research assistant in charge of operating the storefront would ask the participant to use a swab to collect a sample from the inside of their cheek. Samples were then stored in a small, resealable test tube and refrigerated before transfer to −62°C daily after storefront closure.

The dust samples are useful to help understand the participants’ home environment more deeply beyond the HEH survey and what they might be exposed to on campus when attending classes. Examination of the dust samples can determine what types of microbial exposures commonly occur in students’ indoor environments. Buccal swabs can be used for a variety of reasons but were conducted as part of this study to investigate how certain chemical markers such as cytokine levels are related to mood and stress in participants.

### Multiple-time measures

#### Ecological momentary assessment

EMAs involve brief questions about a participant’s behavior and feelings that are answered in real time while the participant is in their natural environment. EMAs were administered using the Beiwe mobile application [15] running on their smartphones at regularly scheduled times throughout each day. For both the Fall 2018 and the Spring 2019 cohorts, EMAs were drawn from 4 categories of questions: sleep questions, momentary context questions, momentary well-being questions, and an audio question. The full text of these questions is provided in Additional File 2. Briefly, the 3 sleep questions were designed to assess the duration and quality of sleep, momentary context questions were designed to determine what the participant was doing and with whom they were doing it, and 5 well-being questions sampled participants’ mood (sadness, loneliness, contentment, and stress) and energy level on a Likert scale. The audio question asked the participant to describe what they were doing and to include a brief segment of background noise.

Both the Fall 2018 and Spring 2019 cohorts received EMAs at 5 different times during the day. At 9:00 each morning they received the 3 sleep questions, 4 momentary context questions that were framed to assess the 15 minutes prior to receiving the EMAs, and 5 mood questions that were framed to assess how they feel at the moment. At 12:00 pm, 3:00 pm, and 6:00 pm participants received EMAs that were similar to those from the morning, except that the sleep questions were removed and the audio question was added. Each night at 9:00 they received the 4 momentary context questions and 5 mood questions that were framed to assess participant behaviors and feelings across the entire day.

### Continuous measures

#### Smartphone

Passive monitoring data were collected using the Beiwe digital phenotyping platform, which is a freely available open-source system that includes mobile phone applications for Apple iOS and Google Android operating systems, and a back-end server implemented in Python. The back-end server was run using Amazon Web Services cloud-based computing infrastructure. The back end includes a study administration web application for designing and conducting Beiwe studies and monitoring their progress, an API for sending study parameters to and receiving data from the mobile phone applications, a database for storing study state information, and an encrypted Amazon Simple Storage Service bucket for storing study data. The Beiwe developers maintain versions of the app in the Apple App store and Google Play store for easy deployment to study participants.

The full set of passive monitoring parameters are provided in Additional File 3. Owing to differences in operating system security settings and device capabilities, different data sources were collected on Android and iOS devices. There were 195 out of the total 1,584 participants who were Android users. Basic device and operating system information (make, model, version), accelerometer, GPS, and power state data were available and collected on both devices. iOS-specific data sources include gyroscope, magnetometer, the proximity of the device to the user, and whether the phone is connected to the internet by WiFi or cellular. Android-specific data sources include a list of WiFi routers and Bluetooth devices in the phone’s proximity; the time, duration, and hashed phone numbers for incoming and outgoing calls; and time, message length, and hashed phone numbers of incoming and outgoing text messages. To maintain participant privacy, WiFi and Bluetooth identifiers, and telephone numbers are encoded with a hashing function. The function is unique, however, so calls to the same destination and proximity to the same WiFi access points can be tracked across time.

The Beiwe mobile application was configured to store collected information locally and to upload it only when connected to the internet using WiFi. If an error was encountered during transmission, the app stores the data and retries transmission until receiving an indication that the data were successfully received by the back end. Each data source is stored in its own set of CSV files that are broken down and organized by timestamp. These files are encrypted on the phone before being transmitted over an SSL connection to the back end. When received, the data are unencrypted, processed to correct for errors, and update received data statistics, and then re-encrypted for storage. All encryption is performed using randomly generated participant-specific encryption keys.

As per the study design, participants were instructed to download and allow all permissions for the Beiwe platform. Each participant had a randomly generated identification tag that consisted of 8 letters and numbers and was prompted to create their own password after entering a temporary password given to them by the study coordinator. Participants did not have direct access to their data and used the login credentials when completing the EMA surveys.

#### Wearable activity tracker

Participants’ activity and sleep patterns were captured using the Fitbit Charge2^TM^ wearable activity trackers. The devices require participants to input their height, weight, gender, and age to accurately calculate the number of steps taken, calories burned, and the wearer’s heart rate. In addition, participants can track different exercises by selecting them from the device’s interface or through the paired smartphone application. Most Fitbit products, including those supplied to the study participants, are capable of passively monitoring the wearer’s sleep as long as the device detects that the user has been asleep for a minimum number of hours. The wearer’s sleep is subdivided into 4 categories based on movement and heart rate: awake, light, deep, and rapid eye movement (REM). Over the past few years, many studies have looked at the accuracy and utility of using Fitbit and other personal monitoring devices in sleep studies [16–18]. Results from these studies show that these devices can be useful when determining total sleep time, awake time, and the amount of time spent in REM sleep.

Similar to the swabs, each Fitbit was numbered, barcoded, and provided to participants also at the storefront location if they consented to participate in the activity-monitoring component of the study. In addition to receiving the device, each participant was required to register a Fitbit account and download the smartphone application if they did not already hold an account. Participants were asked to wear the activity monitors as much as possible, removing them only when bathing, participating in aquatic activities, or charging the device. Participants were instructed to wear the Fitbit monitor over a period of ≥2 weeks and were free to use the device outside of the study requirements. If it was broken or damaged, participants were given a new device to register and use for the remaining portion of the study.

#### Building environment and occupancy beacon

**Figure 2:**
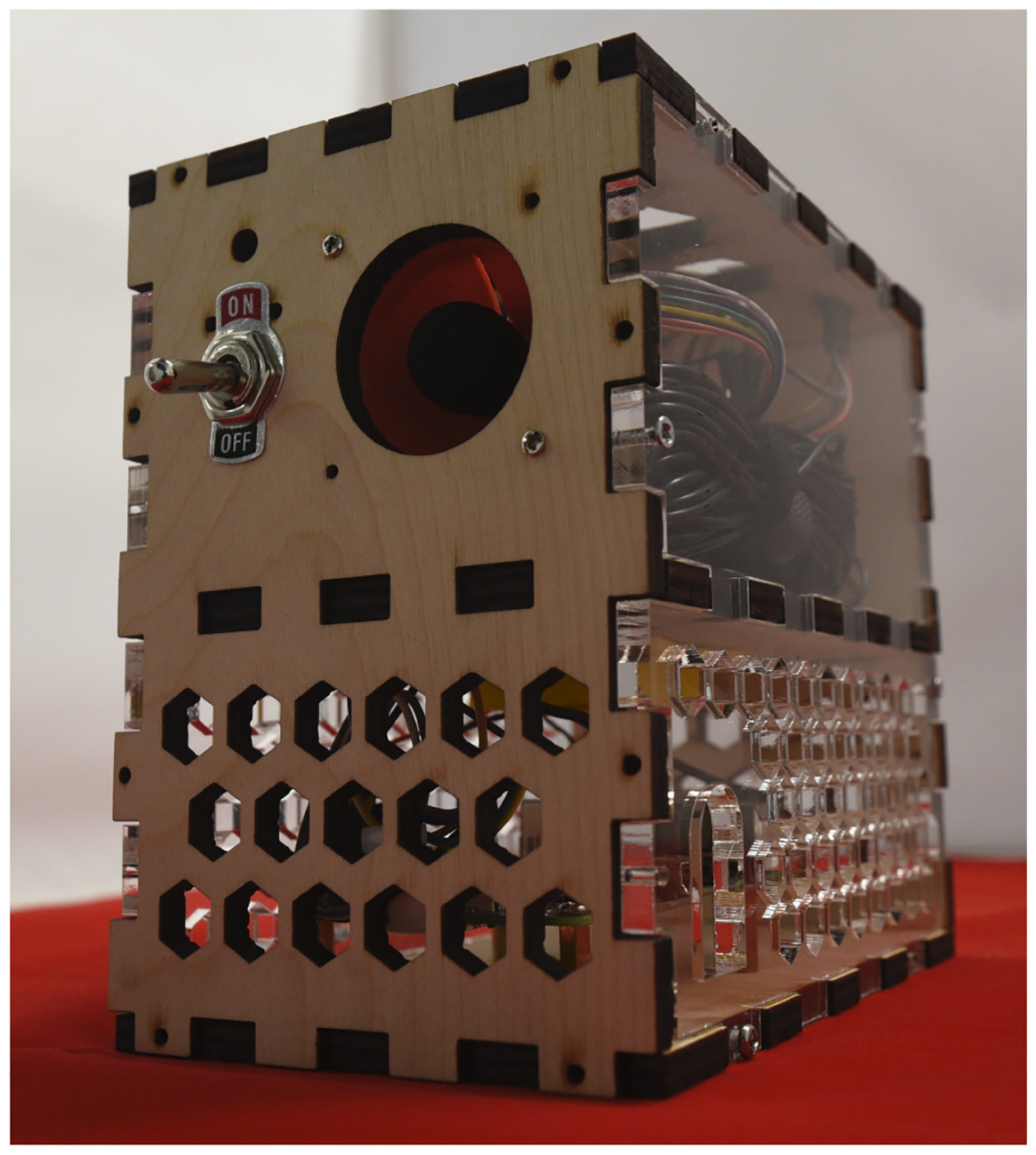
Building EnVironment and Occupancy (BEVO) Beacon.

The Building EnVironment and Occupancy (BEVO) beacon is a low-cost sensor platform that we developed in-house that is capable of measuring multiple indoor environmental quality (IEQ) variables in addition to detecting Bluetooth and WiFi signals. The BEVO Beacon consists of a Raspberry Pi (RPi) 3B+ microcomputer connected to a variety of environmental sensors arranged into a 15 × 15 × 10 cm housing made of plywood and acrylic (see Fig. 2). The RPi can detect Bluetooth devices and WiFi access points in its proximity, which can help determine occupancy. The RPi is additionally capable of storing data captured by itself and the co-locating IEQ sensors on a local micro SD card, and then uploading the data to a cloud-based storage system hosted by the Texas Advanced Computing Center once connected to WiFi. Table 1 outlines the environmental sensors used in BEVO Beacon and the variables that they measure. The RPi is housed in a lower compartment, separated from the sensors and wired to a small fan to help with heat regulation. To avoid the possibility of re-sampling air trapped inside the device’s housing, and further help with heat management, the environmental sensors are housed in a compartment above the RPi with their inlets/exhausts exposed to the ambient air. The BEVO Beacon requires a 5B micro-USB portal connection to power. See Additional File 4 for a detailed 3D sketch of the BEVO Beacon and a full list of the electronic components enclosed.

**Table 1:** IEQ variables measured by the environmental sensors housed in the BEVO Beacon

Variable	Unit	Sensor	Notes
TF	°F	Adafruit SHT31-D	Occupant thermal comfort
RH	%	Adafruit SHT31-D	Occupant thermal comfort
PM	μg/m^3^	Plantower PMS5003	EPA-specified criteria air pollutant with respiratory health implications
TVOCs	ppb	Adafruit SGP30	Compounds whose exposure is related to various health implications such as respiratory issues and cancer

EPA: Environmental Protection Agency; ppb: parts per billion; PM: particulate matter; RH: relative humidity; TF: temperature in Fahrenheit; TVOCs: total volatile organic compounds.

The commercially available sensors used on the BEVO Beacon afforded us 2 benefits. These sensors were low in cost (less than US$100), which allowed us to develop and deploy more devices than typically done when measuring indoor air quality. The second benefit is that these sensors serve as the base units in many other commercially available IEQ products. Using the base units rather than off-the-shelf devices ensures that there is no proprietary algorithm that alters the raw values measured by the sensors. However, reliability and accuracy are 2 main issues surrounding the use of low-cost sensors because they use less sophisticated electronics than high-grade reference monitors that can cost more than US$10,000. Our beacon development effort is a step toward integrating low-cost sensors to achieve high home-sensing performance.

BEVO Beacons were each assigned a number and a barcode. Only the participants who completed the HEH survey were eligible to receive a BEVO Beacon. Participants who were eligible and consented to participation were instructed to stop by the storefront and check out the device. At check-out, the device was supplied to the participant along with a 5V micro-USB to wall outlet adapter. Participants were instructed to power the device using any open outlet in their home. In total, we distributed 15 BEVO Beacons: 5 in Fall 2018 and 10 in Spring 2019.

## Data Validation

### Data collected

**Figure 3:**
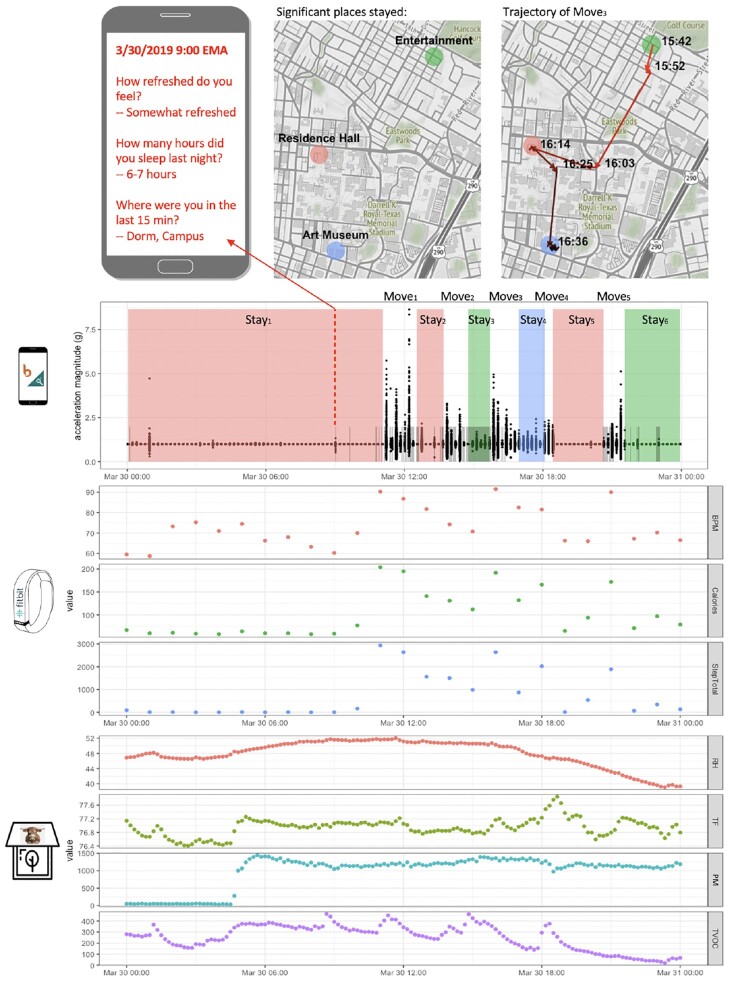
Data collected from the smartphone, Fitbit, and BEVO Beacon of an example participant during a given day (30 March 2019). Plotted data modalities are EMA (questions and answers shown against phone image background on top), GPS (clustered significant places and an example movement trajectory shown in maps on top and as vertical bands), accelerometry (black dots), screen activity (short grey bands), heart rate (BPM), calories spent in the past hour (Calories), steps taken in the past hour (StepTotal), home relative humidity (RH), home temperature in degrees Fahrenheit (TF), home particulate matter in μg/m^3^ (PM), and home TVOC (TVOC).

To showcase the diverse data streams collected from our participants, we plot in Fig. 3 data collected from a particular participant’s smartphone, Fitbit, and BEVO Beacon on a given day (30 March 2019) as an example. The top one-third of Fig. 3 shows the data types collected by the Beiwe smartphone platform. We conducted temporal clustering [19] with the raw GPS traces and processed them into periods of stay at significant places (represented by the colored vertical bands) and periods of movement between significant places (represented by the white spaces between the colored bands). We used Open Street Map API to query for the place type of the significant places found. We show the geographic location and venue type of the significant places, as well as the trajectory and time of the third transition period of the day (“Move_3_”) in the 2 maps on top. In total on 30 March 2019 the participant made 6 stays at 3 distinct places (residence hall, art museum, and entertainment venue) and made 5 trajectories of movement between them. The participant spent her entire night and most of the morning (midnight to ∼11:00 am) at the residence hall (red band/dot), which was her main residence. Notice that even though the trajectory of Move_3_ (top right map) passes through the residence hall it did not register another period of stay, suggesting that the participant merely swung by the residence hall without making a stop for an extended period of time.

During the 3-week official study period, we were able to record some GPS [20] and accelerometer data [21] from ∼950 participants each day. Within these days, data completeness for GPS and accelerometer followed a highly regular and well-synced daily cycle where the least percentage of participants submitted data in the early morning hours and the highest during the evening hours. Averaging the hourly completeness percentages gives us a daily average of ∼65% of the participants submitting data during any given hour. In addition to mobility information, smartphone acceleration magnitude is plotted as black dots and episodes of unlocked screen as short grey bands. We observe that periods of high acceleration magnitude correspond well with periods of movement between places. Screen activity [22], on the other hand, varies heavily depending on the place: e.g., the screen stayed unlocked during the entire Stay_3_ at the entertainment venue but locked during Stay_4_ at the art museum. Moreover, the participant responded to an EMA survey at 9:00 am [23], providing a self-report of her sleep quality and hours of sleep, as well as the semantics of her location at the moment. The participant did not respond to any EMA questions scheduled at other times of the day. Her answer “dorm; campus” to the location question matches with the significant place detected by GPS.

The middle one-third of Fig. 3 shows 3 data streams recorded by Fitbit [24]: heart rate (BPM), calories expended (Calories), and steps taken (StepTotal). The patterns of fluctuation of the 3 data streams are largely in sync with one another, with values significantly higher during the day than during the night. Three points of peak values in heart rate and calories (11:00 am, 4:00 pm, 9:00 pm) visibly correspond to time intervals of high smartphone accelerometer readings, suggesting a positive correlation between smartphone accelerometry and physical activity status recorded by wearable devices. The bottom one-third of Fig. 3 shows 4 data streams recorded by BEVO Beacon [24], all of which are metrics of indoor air quality. Note that only when the participant is located at her home (indicated by red band in this plot, specifically Stay_1, 2, 5_) is she exposed an environment described by these metrics; when she goes away, the metrics merely reflect her home environment status that does not affect her directly. We observe a sharp rise in humidity and PM concentration between 4:00 and 5:00 am, which could indicate a change in the HVAC system.

### Data quality

We evaluate our data’s quality by 2 measures of completeness: first, the amount of time each participant stays in the study and continues to submit data, actively and passively; second, when the data type is continuous, the proportion of time during the entire period of participation that data are available. The first metric is important because it represents participant adherence and a higher value in it (or, closer to the length of the intended study period) indicates more successful participation. The latter measure is also important in that it represents data continuity and a higher value in it indicates fewer data-missing intervals during the total period of time a participant is submitting data.

**Figure 4:**
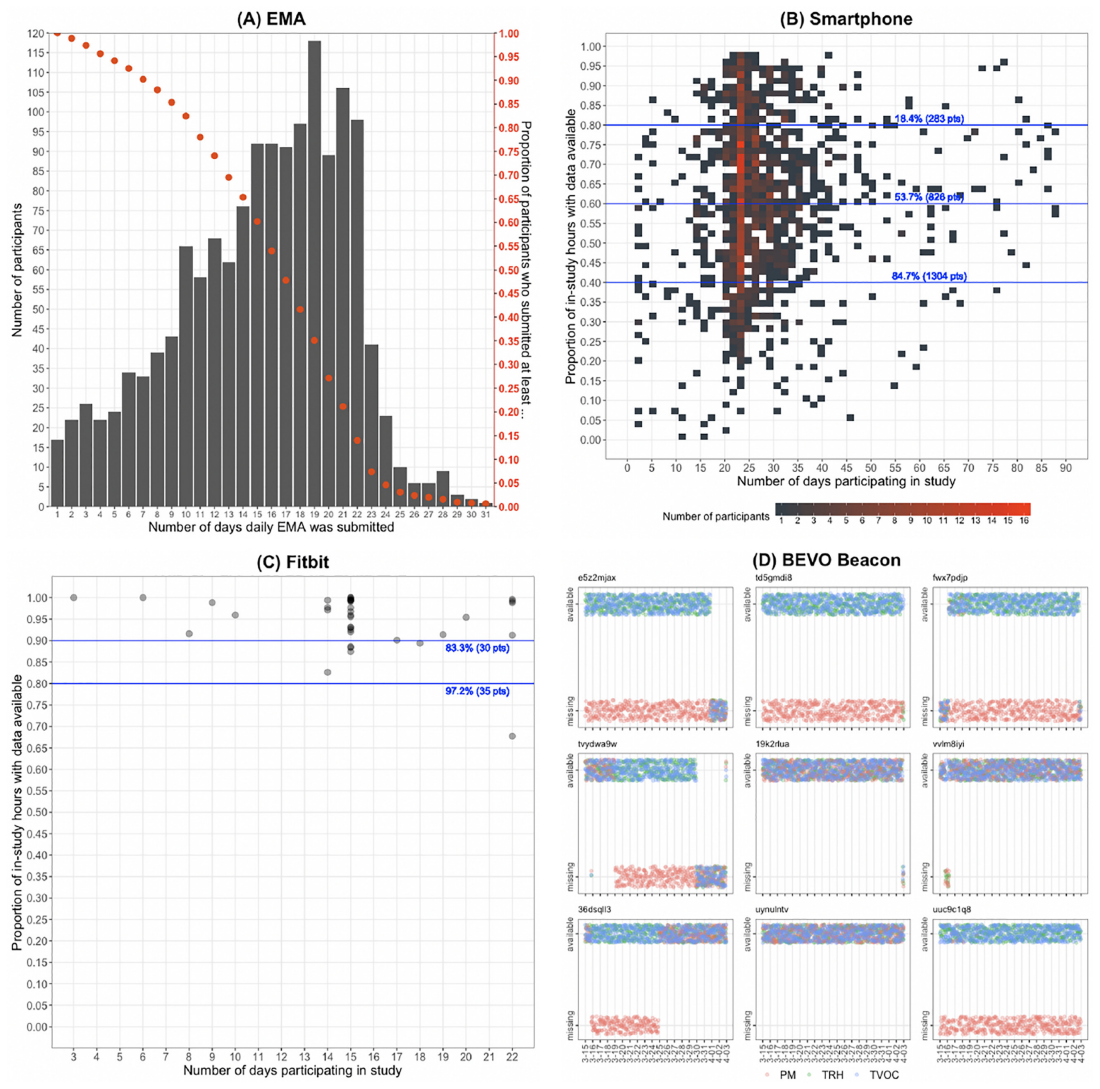
Completeness of 4 types of data collected from participants in the Fall and Spring deployments combined: (A) daily EMA (1,482 participants submitted data); (B) smartphone sensing, GPS data shown as example (1,539 participants); (C) Fitbit (36 participants); and (D) BEVO Beacon (9 participants).

In Fig. 4 we present the completeness of 4 major types of data that we collected, namely, EMA, smartphone, Fitbit, and BEVO Beacon. In Fig. 4A, we show the number of participants (height of bars) who submitted answers to daily EMAs for all different numbers of days (horizontal position of bars), as well as the percentage of participants (projection of red dots on vertical axis on right) who submitted answers to daily EMAs at least a certain number of days (horizontal position of red dots). We observe that >60% of participants submitted daily EMA answers for >14 days and >20% for >21 days.

In Fig. 4B, we show the distribution of participants with respect to the total duration of time that they were contributing smartphone GPS data (i.e., adherence, on the horizontal axis) and the proportion of that duration for which their data are available (i.e., continuity, on the vertical axis). We use the color of square cells to indicate the number of participants who fall in particular compliance-continuity boundaries: the brighter-colored vertical bar between 20 and 25 days corresponds well with our planned study length, which is 3 weeks. Shown by the blue horizontal lines, 283 (18.4%) participants had smartphone GPS data available for >80% of the hours they participated in study, 826 (53.7%) participants >60%, and 1,304 (84.7%) participants >40%.

Fig. 4C contains the same information as 4B but plotted for Fitbit data. As opposed to aggregating the number of participants like we did for 4B, because of the lower number of participants who chose to wear a Fitbit (36), we simply represented each Fitbit participant as an opaque grey dot, forming a darker cluster when more participants fall in a close region of compliance-continuity value combination. Compared to smartphone data, Fitbit data displayed significantly higher continuity: of the 36 participants, 30 submitted data for >80% of their in-study time. This may suggest that Fitbit as a wearable device requires less human attention and interference, as opposed to smartphones, which are constantly being handled and require more frequent charging, and thus are more prone to produce uninterrupted data streams.

Of the 15 BEVO Beacons we distributed, we found that only 9 recorded and uploaded data reliably and they were all from the Spring 2019 deployment. Owing to such a small number of participants who returned substantial environmental sensing data, in Fig. 4D we simply plotted entire time series of data availability of each IEQ measure captured by BEVO Beacons (indicated by different colored dots, jittered) for each of the 9 participants. Visibly, 3 of the 9 BEVO Beacons submitted data perfectly whereas the remaining 6 primarily had trouble submitting PM data (red strip on the bottom). Upon inspection we found that communication errors between the sensors and the RPi accounted for a large amount of data loss. Fluctuations in power delivery between the RPi and the environmental sensors caused some environmental sensors to go offline for periods and resulted in data loss during the study.

One highly notable difference is in the number of participants who signed up for smartphone sensing compared to wearable and home environment sensing (>1,000 vs <100). We believe that both hardware availability and the incentive structure contributed to this discrepancy. First, the smartphone component of the study required the participants’ own primary phone, which was widely available; whereas the Fitbit and BEVO Beacon components required extra hardware that we needed to purchase, build, and provide for the participants, thus limiting the number of participants we could enroll. Second, the incentive for students to complete smartphone sensing and EMAs was to receive credits on an assignment that counted toward their final grade (with the alternative to opt out of using smartphone and only logging manually instead), which proved to be an effective strategy to improve participant adherence [25]; whereas participation in the other parts of the study was rewarded by extra experimental credits, which may not have been as attractive to the students.

## Reuse potential

A primary advantage of our collected data is the large number of college students recruited as participants. With comprehensive momentary self-reports covering mood, type of place, social context, and activities of daily living, one can gain a representative picture of the daily experience of college students at a major university in North America. Interrelations between different aspects of mood as well as between mood and place, social context, and the concurrent activities of daily living may be of value to researchers interested in college students’ well-being. Moreover, the large amounts of smartphone GPS data can serve as evidence for the temporal-geographic distributions of students’ campus usage, which may be of interest to crowd-sensing researchers.

Besides examining individual data types, we believe that our data offer many opportunities for analyses focusing on cross-modality correlations. This can be done between 2 separate streams of sensing data or between sensing data and self-reported variables from momentary and pre-study surveys. A potential purpose of the between-sensors comparison is to ascertain their respective sensing capabilities of similar constructs, e.g., between a smartphone’s accelerometer and a Fitbit’s step count and heart rate measurements. Additionally, jointly utilizing ≥2 sensing data streams creates opportunities for novel analytical methods that help understand and predict human well-being outcomes more effectively [26]. In the case of sensor-survey comparison, a fundamental research question is whether and to what extent do variances in the sensing data correlate with variances in the self-reported data, which typically reflect the ground truth about a participant’s health and behavioral states. There have been numerous digital phenotyping studies on using various mobile sensing data streams to detect mental health abnormalities and fluctuations. Our data may serve as a testbed for researchers wishing to validate different feature-engineering and data-mining techniques in terms of their contributions to predictive performance.

A shared characteristic of the different data types in our collection is having multiple measurements from the same participant, which may have a broader relevance to statistical analysis in human-subject studies. Typically, when researchers collect experimental data from participants, multiple data points for each data type are collected from each participant, resulting in a situation where one has *N* observations from *M* participants and *N* > *M*. This is not a trivial issue because observations that belong to 1 participant tend to be more correlated with each other than with those from other participants, which makes the direct application of regression models onto the entirety of the *N* observations statistically unsound [27]. If the inter-individual variation in a particular variable is of interest and the number of participants *M* with data available is satisfactorily high, 1 appropriate approach is to aggregate the variable by the individual, such that each individual is mapped to only 1 value (e.g., mean value) of that variable. Interpretations made from models created on such aggregated data would be regarding the cross-participant patterns rather than within-participant ones. If one has a limited amount of data available that does not justify aggregation, an appropriate solution is to use mixed-effect models to account for individual differences between participants. An individual random effect could be added to both the intercept or a variable coefficient of a (generalized) linear model, and the eventual selection will need to depend on model selection metrics or tests such as Akaike information criterion, Bayesian information criterion, or a likelihood ratio test to determine whether the added effect (thus increasing variance explained) is worth the increased model complexity.

## Conclusion

We conducted the UT1000 Project, a multi-modal data collection study using a variety of technologies and methods to monitor and understand aspects of the health, behavior, and living environments (see Fig. 3) of a college student cohort of >1,500 participants for 3 weeks. Some participants voluntarily monitored themselves for several more weeks after the official 3-week study period ended. The project is novel due to not only the large scale of participation but also the emphasis on incorporating the monitoring of personal living environment with health and behavior sensing to achieve a multi-faceted dashboard of human-centric information. With many types and sources of data at hand, we proposed a conceptual framework systematically organizing human-centric data modalities and their corresponding technologies and methods with respect to their temporal coverage and spatial freedom, which is further helpful for guiding data collection and research question formulation. Temporal coverage and spatial freedom overlap with ecological validity and are constrained by the unobtrusiveness of the method. Hurdles to unobtrusiveness include the size, weight, and power need of a device, requirement for human attention and maintenance, and many potential others. A general direction of evolution for human-centric design and technology is to become more portable, convenient, and user-friendly thus affording higher and higher unobtrusiveness and ecological validity for its capacity of understanding and assisting humans, until it is truly “woven into the fabric of everyday life” [28].

We were able to collect from a large participant cohort satisfactorily complete multi-modal data in terms of both data continuity and participant compliance (see Fig. 4). Collection of multi-modal high-density datasets from humans in the field, like that presented in this study, has unique challenges. At enrollment, it is important to reassure potential participants that the data being collected are secure and their privacy will be respected and maintained. In the undergraduate sample used here, this did not seem to be a big concern for the students. Roughly 3–4% of students cited data privacy as a reason they did not want to participate. A second issue encountered was the compatibility of the smartphone application Beiwe with various software versions of iOS and Android and models of Apple and Android-compatible phones. Most devices worked well, but a noticeable percentage did not. It was not always obvious what determined whether the software would work with a particular device and the study did not have the resources, nor focus for a systematic evaluation of these issues. An additional challenge was data fidelity. When collecting continuous data from multiple devices (smartphones, Fitbits, Beacons), data transfer to a database was subjected to issues of connectivity, bandwidth, and participant adherence (keeping the device charged, connecting to WiFi regularly, and s forth). Finally, data wrangling and clean-up is particularly challenging. The data being collected are on multiple time scales; with missing data scattered throughout, methods of data imputation and alignment create major decision points.

Several limitations exist in our study, which we would like to address in future work. First, we would like to monitor participants for a longer period than 3 weeks so that we can observe more reliable patterns of behavioral variation and build more accurate personalized predictive models. Second, there is a sharp imbalance between the availability of smartphone and EMA data and the availability of Fitbit and BEVO Beacon data due to our incentive structure and hardware availability. We are preparing for a future deployment of the UT1000 Project that directly addresses these limitations by recruiting (potentially fewer) participants who will commit to longer study periods as well as increasing the number of Fitbits and BEVO Beacons distributed. Another type of data that we did not collect in the UT1000 Project is individual social media data including both the content created and the interaction patterns, which have been utilized to predict personal mental health outcomes in recent studies [29]. In our conceptual framework of human-centric data modality, social media data would fit in the medium range on both the temporal coverage and the spatial freedom axes, similar to the position of EMAs. We anticipate the collection, integration, and mining of diverse modalities of human-centric data from different technologies and methods and of various degrees of temporal coverage and spatial freedom to be key to the development of a new generation of digital solutions for personal well-being enhancement.

## Data Availability

The datasets supporting the results of this article are available in the “UT1000 Data for MADS Paper” repository at https://dataverse.tdl.org/dataverse/ut1000_mads. In the repository, different data types are compiled in separate datasets as follows:

EMA [23];

Accelerometer [21];

GPS [20];

Screen unlock events [22];

Fitbit/BEVO Beacon [24]; along with a participant key file provided showing our participants’ gender, age, and year of participation (2018 or 2019) [30].

## Additional Files

Additional File 1: HEH Survey Questions

Additional File 2: EMA Questions

Additional File 3: Beiwe Sensing Parameters

Additional File 4: A detailed 3D sketch of the BEVO Beacon and a full list of electronic components enclosed

## Abbreviations

API: application programming interface; BEVO: Building EnVironment and Occupancy; EMA: ecological momentary assessment; GPS: Global Positioning System; HEH: home environment and health; HVAC: heating, ventilation, and air conditioning; IEQ: indoor environmental quality; PM: particulate matter; REM: rapid eye movement; RH: relative humidity; RPi: Raspberry Pi; SSL: Secure Sockets Layer; TF: temperature in Fahrenheit; TVOC: total volatile organic compounds; USB: Universal Serial Bus.

## Ethical Approval

The study described in this article was approved by the University of Texas Institutional Review Board (study No. 2018-07-0035; approval date: 25 September 2018; expiration: 16 July 2019).

## Consent for Publication

Consent for presenting de-identified individual data was obtained from participants before study started. Consent form is available upon request.

## Competing Interests

The authors declare that they have no competing interests.

## Funding

This work was supported by Whole Communities—Whole Health, a research grand challenge at the University of Texas at Austin, and National Science Foundation Award SES-1758835.

## Author’s Contributions

C.W.: Conceptualization, Data curation, Formal Analysis, Methodology, Project administration, Software, Validation, Visualization, Writing—original draft, Writing—review and editing.

H.F.: Conceptualization, Data curation, Formal Analysis, Methodology, Resources, Software, Validation, Visualization, Writing—original draft.

S.B.: Conceptualization, Data curation, Methodology, Resources, Visualization, Writing—original draft.

J.P.M.: Conceptualization, Data curation, Methodology, Resources, Writing—original draft.

E.T.: Conceptualization, Data curation, Methodology, Writing—review and editing.

C.J.: Conceptualization, Data curation, Methodology, Writing—review and editing.

D.M.C.: Conceptualization, Data curation, Methodology, Writing—review and editing.

K.B.: Conceptualization, Data curation, Methodology, Writing—review and editing.

S.K.B.: Conceptualization, Data curation, Methodology, Writing—review and editing.

G.M.H.: Conceptualization, Data curation, Funding acquisition, Methodology, Resources, Writing—review and editing.

R.C.C.: Conceptualization, Data curation, Funding acquisition, Methodology, Project administration, Resources, Supervision, Visualization, Writing—original draft, Writing—review and editing.

K.A.K.: Conceptualization, Data curation, Funding acquisition, Methodology, Project administration, Resources, Supervision, Visualization, Writing—review and editing.

S.D.G.: Conceptualization, Data curation, Funding acquisition, Methodology, Resources, Supervision, Visualization, Writing—review and editing.

D.M.S.: Conceptualization, Data curation, Funding acquisition, Methodology, Project administration, Resources, Supervision, Visualization, Writing—original draft, Writing—review and editing.

Z.N.: Conceptualization, Data curation, Funding acquisition, Methodology, Project administration, Resources, Supervision, Visualization, Writing—original draft, Writing—review and editing.

## Supplementary Material

giab044_GIGA-D-20-00353_Original_Submission

giab044_GIGA-D-20-00353_Revision_1

giab044_GIGA-D-20-00353_Revision_2

giab044_Response_to_Reviewer_Comments_Original_Submission

giab044_Response_to_Reviewer_Comments_Revision_1

giab044_Reviewer_1_Report_Original_Submission Aku Visuri -- 1/3/2021 Reviewed

giab044_Reviewer_2_Report_Original_Submission Thomas E. Doyle, Ph.D., M.E.Sc., B.E.Sc., B.Sc. -- 2/16/2021 Reviewed

giab044_Supplemental_Files
