# Supplementary material for: Multi-modal data collection for measuring health, behavior, and living environment of large-scale participant cohorts
Source: Gigascience. 2021 Jun 21;10(6):giab044. doi: 10.1093/gigascience/giab044 (PMC8216865; doi:10.1093/gigascience/giab044)
Supplement: giab044_GIGA-D-20-00353_Revision_1 [file giab044_giga-d-20-00353_revision_1.pdf]

GigaScience

Multi-Modal Data Collection for Measuring Health, Behavior, and Living Environment of Large-Scale Participant Cohorts

--Manuscript Draft--

**Manuscript Number:**	GIGA-D-20-00353R1
**Full Title:**	Multi-Modal Data Collection for Measuring Health, Behavior, and Living Environment of Large-Scale Participant Cohorts
**Article Type:**	Data Note
**Funding Information:**	National Science Foundation (SES-1758835) — Dr. Samuel D. Gosling<br>Whole Communities—Whole Health, a research grand challenge at the University of Texas at Austin — Not applicable
**Abstract:**	Background As mobile technologies become ever more sensor-rich, portable, and ubiquitous, data captured by smart devices are lending rich insights into users' daily lives with unprecedented comprehensiveness and ecological validity. A number of human-subject studies have been conducted to examine the use of mobile sensing to uncover individual behavioral patterns and health outcomes, yet minimal attention has been placed on measuring living environments together with other human-centered sensing data. Moreover, the participant sample size in most existing studies falls well below a few hundred, leaving questions open about the reliability of findings on the relations between mobile sensing signals and human outcomes. Results To address these limitations, we developed a home environment sensor kit for continuous indoor air quality tracking and deployed it in conjunction with smartphones, Fitbits, and ecological momentary assessments in a cohort study of up to 1584 college student participants per data type for three weeks. We propose a conceptual framework that systematically organizes human-centric data modalities by their temporal coverage and spatial freedom. Then we report our study procedure, technologies and methods deployed, descriptive statistics of the collected data that reflect the participants' mood, sleep, behavior, and living environment. Conclusions We were able to collect from a large participant cohort satisfactorily complete multi-modal sensing and survey data in terms of both data continuity and participant compliance. Our novel data and conceptual development provide important guidance for data collection and hypothesis generation in future human-centered sensing studies.
**Corresponding Author:**	Congyu Wu<br>University of Texas at Austin<br>Austin, TX UNITED STATES
**Corresponding Author Secondary Information:**	
**Corresponding Author's Institution:**	University of Texas at Austin
**Corresponding Author's Secondary Institution:**	
**First Author:**	Congyu Wu
**First Author Secondary Information:**	
**Order of Authors:**	Congyu Wu<br>Hagen Fritz<br>Juan P. Maestre<br>Edison Thomaz<br>Christine Julien<br>Darla Castelli<br>Kaya de Barbaro

Powered by Editorial Manager® and ProduXion Manager® from Aries Systems Corporation

	Sarah Kate Bearman
	Gabriella Harari
	Cameron Craddock
	Kerry Kinney
	Samuel D. Gosling
	David M. Schnyer
	Zoltan Nagy
**Order of Authors Secondary Information:**	
**Response to Reviewers:**	Dear Hans,<br><br>In keeping with our recent email exchanges, we have converted our manuscript to a Data Note. We kept most of the original content and sections as they align quite well with the requirements of a Data Note. We removed some research question driven analyses and added a Re-use Potential section. We believe now the manuscript is in a good shape for a Data Note publication. We appreciate the valuable guidance you've been offering along the way.<br><br>Sincerely,<br>Congyu
**Additional Information:**	
**Question**	**Response**
Are you submitting this manuscript to a special series or article collection?	No
**Experimental design and statistics**<br><br>Full details of the experimental design and statistical methods used should be given in the Methods section, as detailed in our Minimum Standards Reporting Checklist. Information essential to interpreting the data presented should be made available in the figure legends.<br><br>Have you included all the information requested in your manuscript?	Yes
**Resources**<br><br>A description of all resources used, including antibodies, cell lines, animals and software tools, with enough information to allow them to be uniquely identified, should be included in the Methods section. Authors are strongly encouraged to cite Research Resource Identifiers (RRIDs) for antibodies, model	Yes

Powered by Editorial Manager® and ProduXion Manager® from Aries Systems Corporation

organisms and tools, where possible.<br><br>Have you included the information requested as detailed in our Minimum Standards Reporting Checklist?	
**Availability of data and materials**<br><br>All datasets and code on which the conclusions of the paper rely must be either included in your submission or deposited in publicly available repositories (where available and ethically appropriate), referencing such data using a unique identifier in the references and in the “Availability of Data and Materials” section of your manuscript.<br><br>Have you have met the above requirement as detailed in our Minimum Standards Reporting Checklist?	Yes

Powered by Editorial Manager® and ProduXion Manager® from Aries Systems Corporation

Manuscript

Click here to access/download;Manuscript;Giga_revision_manuscript_highli

OXFORD

(GIGA)^n SCIENCE

GigaScience, 2017, 1–10

doi: xx.xxxx/xxxx
Manuscript in Preparation
Paper

PAPER

Multi-Modal Data Collection for Measuring Health, Behavior, and Living Environment of Large-Scale Participant Cohorts

Congyu Wu[1,*], Hagen Fritz[2], Juan P. Maestre[2], Edison Thomaz[3], Christine Julien[3], Darla M. Castelli[4], Kaya de Barbaro[1], Sarah Kate Bearman[5], Gabriella M. Harari[7], R. Cameron Craddock[6], Kerry A. Kinney[2], Samuel D. Gosling[1,8], David M. Schnyer[1] and Zoltan Nagy[2]

[1]Department of Psychology, University of Texas at Austin and [2]Department of Civil, Environmental, and Architectural Engineering, University of Texas at Austin and [3]Department of Electrical and Computer Engineering, University of Texas at Austin and [4]Department of Kinesiology and Health Education, University of Texas at Austin and [5]Department of Educational Psychology, University of Texas at Austin and [6]Department of Diagnostic Medicine, University of Texas at Austin and [7]Department of Communication, Stanford University and [8]Melbourne School of Psychological Sciences, University of Melbourne

[*]Correspondence: congyu.wu@austin.utexas.edu

Abstract

Background As mobile technologies become ever more sensor-rich, portable, and ubiquitous, data captured by smart devices are lending rich insights into users' daily lives with unprecedented comprehensiveness and ecological validity. A number of human-subject studies have been conducted to examine the use of mobile sensing to uncover individual behavioral patterns and health outcomes, yet minimal attention has been placed on measuring living environments together with other human-centered sensing data. Moreover, the participant sample size in most existing studies falls well below a few hundred, leaving questions open about the reliability of findings on the relations between mobile sensing signals and human outcomes. **Results** To address these limitations, we developed a home environment sensor kit for continuous indoor air quality tracking and deployed it in conjunction with smartphones, Fitbits, and ecological momentary assessments in a cohort study of up to 1584 college student participants per data type for three weeks. We propose a conceptual framework that systematically organizes human-centric data modalities by their temporal coverage and spatial freedom. Then we report our study procedure, technologies and methods deployed, des[illegible]ve statistics of the collected data that reflect the participants' mood, sleep, behavior, and living environment. **[illegible]usions** We were able to collect from a large participant cohort satisfactorily complete multi-modal sensing and survey data in terms of both data continuity and participant compliance. Our novel data and conceptual development provide important guidance for data collection and hypothesis generation in future human-centered sensing studies.

Key words: Multi-modal sensing; human-centered computing; smartphone; Fitbit; BEVO Beacon; ecological momentary assessment; health; college students

Compiled on: March 26, 2021.
Draft manuscript prepared by the author.

1 Introduction

Human health and behavioral research is primarily conducted in laboratories under conditions that poorly approximate real-world conditions. While this model has been successful, it may miss key aspects of human behaviors that are elicited only during more natural conditions or interactions. This concern has driven interest in developing and using remote sensing technologies to measure individuals completing their normal day-to-day activities in their natural environment. An explosion in modern technologies, many of which are in common usage, now provide the ability to monitor and understand health and human behavior in ways not previously possible. Smartphones, smart home devices, wearables, and online digital behaviors provide new ways to track sleep, emotions, spatial mobility, activity, environmental exposures, and social interactions to name just a few. These technological advances offer opportunities to unobtrusively collect real-time data on a wide range of social-behavioral and health variables with less participant burden and more ecological validity than ever before [1]

In the past decade we have seen growing effort worldwide in collecting real-time sensing and experience sampling data from human participants in natural, uncontrolled settings [2, 3, 4, 5, 6, 7]. Smartphones are the staple of sensing hardware to measure different aspects of daily behavior [8]. Four main categories of behavioral patterns can be captured passively: (a) mobility trajectories, measured by GPS and further processed into location clusters that represent significant places visited [9]; (b) physical activity, measured by accelerometer and further processed into activity status labels such as walking and staying still [10]; (c) social context, reflected in different modes including phone calls, message exchange, and physical proximity detected by Bluetooth, which may be used to reconstruct social networks [11], and; (d) interaction with the device, such as screen unlock status and app usage, which are logged by the smartphone itself [12]. Additionally, ecological momentary assessment (EMA) surveys can be deployed to actively collect participant's self-reports of mood, behavior, and well-being in real time.

While many aspects of behavior and health can be captured in smartphone sensing data including EMAs with satisfactory ecological validity, we realize that other key dimensions of well-being are better measured using complementary technologies. A person spends a significant proportion of their time at home but measurements of their home environment are not generally investigated in existing studies in parallel with other aspects of daily behavior. To this end, we developed a home environment sensing device, named *BEVO Beacon*, that is capable of continuously collecting and uploading multiple measures of indoor air quality. This device can provide critical insights into a participant's living environment and evaluate its behavioral and health implications. Additionally, sleep is a critical health outcome and independent variable that is difficult to measure objectively using unobtrusive instruments. We argue it is especially beneficial to utilize the sleep measuring capability of wearable devices such as Fitbit to validate EMA answers.

Furthermore, the vast majority of existing human sensing studies used less than a few hundred participants [13]. A larger sample is needed to obtain more reliable assessments of the correlations between key behavior and health measures, especially when a large number of variables is assessed. Reflecting these considerations, we conducted a multi-modal human sensing study named *UT1000*, for which we recruited more than 1000 college students as participants over two deployments and distributed a variety of sensors and instruments including smartphone, Fitbit, BEVO Beacon, and EMA. The resulting data allow us to pursue research questions that previous data were unable to accommodate.

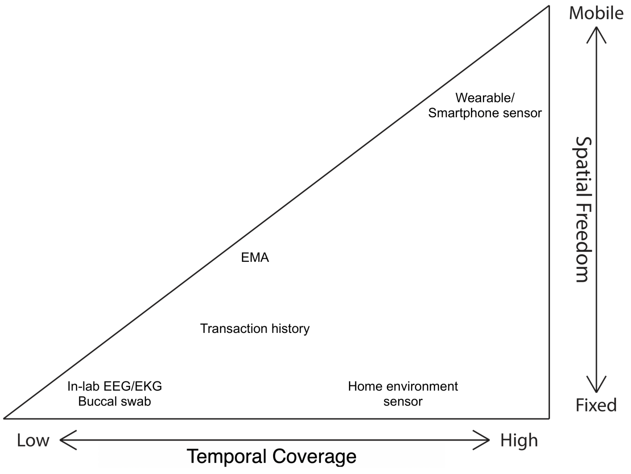

Figure 1. Human-centric Data Modality Framework

With numerous types of technologies and methods potentially available to measure individual humans' health, behavior, and environment, we begin in Section 2 by proposing a novel conceptual framework that organizes the various modalities of human-centric data based on their properties. Then, we present the study design and the types of data collected in our UT1000 Project in Section 3. We visualize the concurrent data streams collected during the study and evaluate their completeness in Section 4. In Section 5 we outline potential analytical and research applications for which our data can be useful. Through this study we are able to gain comprehensive understandings of the lives of college students and learn valuable lessons about the design, deployment, and data analysis for large-scale human sensing studies.

2 Conceptual Framework

Figure 1 illustrates the conceptual framework we devised for organizing different technologies and methods for observing human outcomes based on properties of their data collection procedure and resulting data.

A primary property of human-centric data is its *temporal coverage*, represented by the bottom horizontal axis from low (left) to high (right). Temporal coverage is defined by the inherent suitability of a data modality to monitor extended proportions of time of an individual's daily life. Data modalities that can provide only single-time observations are on the low end of the temporal coverage dimension. Examples include (1) traditional survey inventories that are designed to provide a one-time diagnosis of a potential patient and (2) medical procedures that typically require in-person clinical visits such as electroencephalography (EEG), electrocardiography (EKG), and buccal swabs. Some data collection methods or technologies accommodate measurements taken at multiple points in time, thus are placed mid-range along the temporal coverage dimension. Examples include (1) self-reports in response to EMAs delivered via mobile devices and (2) record data such as transaction history which contains user information logged at different times of user engagement with a service or services. Highest temporal coverage is achieved by continuous tracking. Various sensors embedded in devices people carry where they go and install where they stay belong in this category, such as smartphones, wearable devices, and environmental sensors used as smart home technology. Wearable devices including smartphones tend to enjoy an even higher level of temporal coverage than environmental sensors. This is because wearable devices are able to accompany and monitor the user at all

times as long as the user keeps the devices on their person and powered on, thus observing the user for longer periods of time compared to environmental sensors, which can only offer *human-centric* sensing capability when the individual in question remains within their proximity (e.g., home environment sensors are only human-centric when the person is at home).

A second property of human-centric data is its *spatial freedom*, represented by the vertical axis ranging from fixed (bottom) to mobile (top). Spatial freedom is defined by the ability of a data modality to reflect an individual's health, behavior, and environment at a variety of locations. Data modalities with greater temporal coverage tend to permit a wider range of locations where an individual can be monitored, a.k.a. higher spatial freedom. For this reason the coordinate space presented in Figure 1 is a triangle. A discrete-time measurement of low temporal coverage is typically taken at a specific location, only allowing minimal spatial freedom, thus occupying the left vertex of the triangle. However, spatial freedom may vary greatly among instruments that can track users continuously over time. For example, both a PM2.5 sensor installed at home and a smart wristband can be considered as having high temporal coverage; however, they correspond to fixed and mobile, respectively, on the spatial freedom dimension.

The temporal coverage and spatial freedom of a data modality is often governed by the unobtrusiveness [14] of the technology or method producing it. Data modalities of higher temporal coverage and spatial freedom are usually produced by devices and procedures that are more user-friendly, more portable, and overall less burdensome for the user. Unobtrusive methods allow for more naturalistic and non-interfering ways to monitor a participant's daily life, thus producing measures of greater ecological validity. We identify a major correlation between ecological validity and temporal coverage/spatial freedom: measures of high ecological validity tend to enable observations over extended periods of time and with greater mobility. However, the relation between ecological validity and temporal coverage/spatial freedom is not a necessary one. In the classic example of Barker & Wright's study of Raymond Birch [15], in which a research team followed a subject around for a whole day making observations every few minutes, temporal coverage and spatial freedom are both high however ecological validity is low, because the observation method was extremely intrusive.

The example data modalities shown in Figure 1 are based on how the generating methods are naturally and realistically carried out. For example, a buccal swab procedure takes a few minutes to complete but it requires a high level of participation and effort from the patient; therefore even though technically buccal swabs can be frequently administered, we still consider it to be a highly intrusive, one-time measurement. Many human-monitoring technologies, over their course of development, have seen themselves ascend on the temporal coverage scale and often on spatial freedom as well. For example, blood glucose testing used to require clinical visits, thus the burden was high; however, as the technology for on-body continuous glucose monitoring becomes perfected, blood glucose measurements can be obtained with unprecedentedly high unobtrusiveness, allowing its temporal coverage and spatial freedom to increase as well.

A critical challenge in interdisciplinary research joining social sciences and engineering is the high-fidelity mapping between human-centric constructs and technology-advanced methods. Our framework provides an interface between the constructs and the methods based on their temporal and spatial characteristics and implications for ecological validity and unobtrusiveness. Thus it can help social scientists locate fitting methods to measure the constructs of interest. Furthermore, this framework can be used to guide data collection and hypothesis generation concerning interrelations of different aspects of human behavior. Researchers can delineate a subarea in the triangle to serve as the scope of their own data collection. Hypotheses or research questions can be straightforwardly formulated by linking two spots on the triangle and querying the relationship between the two corresponding data modalities. Moreover, the two-dimensional space describes one individual and can be conceptually stacked up to represent a group of individuals and their data and descriptors. Linkage between slices representing different individuals can inform the generation of research questions into the relations between individual outcomes and inter-personal interactions.

3 The UT1000 Project

The UT1000 Project is a multi-modal data collection study conducted at the University of Texas at Austin to measure aspects of the health, behavior, and home environment of a large-scale participant cohort using a wide variety of technologies and methods, including traditional surveys, swabbing, EMAs, smartphone sensing, wearable trackers, and environmental sensors [1]. We undertook two deployments, one in the Fall of 2018 the other in the Spring of 2019, totaling 1584 participants (62% female) and lasting three weeks each. Participants for this study were recruited through an introductory psychology course. Enrolled students were instructed to sign up for the EMA and smartphone sensing components of the study as a class assignment that counted toward their final grade. Students who did not want to self-track using a smartphone were given the option to record their behaviors and moods by answering emailed EMA questions or keeping a daily diary. The other data modalities such as those from the wearable trackers and environmental sensors, on the other hand, were collected in return for experimental credits which the students used as partial fulfillment of the course requirements. The following subsections outline the different components of the UT1000 Project including the purpose, procedure, and types of data collected. Discussions of different study components are organized into three main categories based on the data modalities, namely *single-time*, *multiple-time*, and *continuous* measures, following an order of temporal coverage from low to high consistent with the horizontal dimension of Figure 1.

3.1 Single-time measures

3.1.1 Home Environment and Health (HEH) Survey

The Home Environment and Health (HEH) questionnaire consists of 63 questions asking students to report on home environment factors such as their current living situation including number of roommates, number and type of pets, and flooring type; their recent health and medical histories including colds, allergies, and flu shots received; and other behaviors such as hand washing frequency and use of electric scooters. A full list of the HEH questions are provided [see Additional File 1]. The purpose of this survey was to obtain a better understanding of the participants' home environment and to clarify discrepancies found in the other data streams.

The HEH questionnaire was a voluntary survey sent directly to participants via the email they provided to register for the study. Completing the HEH survey was a prerequisite for the subsequent home environment sensing component of the study discussed in Section 3.3.3. The survey was sent once during the first two weeks of the study period. Participants were asked to

1 University of Texas Institutional Review Board study number 2018-07-0035; approval date: September 25, 2018; expires: July 16, 2019.

fill out the survey based on their situation when they received it rather than some time in the past or the future. A total of 56 participants completed the HEH questionnaire, with 46 in Fall 2018 and 10 in Spring 2019.

3.1.2 *Student Environment and Buccal Swabbing*

A subset of the study participants were provided with a dust sampling kit to collect dust samples from various surfaces in their home and classroom environment. The same participants that completed the HEH survey were given the kits (N = 56). The kit consisted of six, individually-packaged Phosphate-Buffered Saline Tween-20 (PBST) wetted FLOQswabs® (manufactured by COPAN[2], Murrieta, CA) and six corresponding plastic, resealable test tubes that participants would place the swabs in after collecting samples. Participants were asked for identification in order to gather sampling materials from a refrigerator-equipped storefront created ad hoc in a convenient place at a central location of the university. Testing materials to be distributed to different participants were labeled with distinct barcodes so that we could easily trace the materials back to the participants and streamline the checkout process.

Participants followed instructions to collect samples from the interior and exterior of their front door trim, cellphone screen, living room floor, HVAC filter or air diffuser if applicable, and a desktop where they normally sit when attending university classes. After sample collection, participants sealed swabs in the provided test tubes and placed them in their refrigerator until transportation to the university lab. When participants return the testing materials, material barcodes were scanned, the identity of the participant cross-referenced to the materials, and the temperature-sensitive samples were stored until transfer to a -4°F freezer daily after storefront closure. They were asked to provide feedback on the challenges while performing home sampling and also whether they are willing to submit a buccal swab. If they consented, the research assistant in charge of operating the storefront would ask the participant to use a swab to collect a sample from the inside of their cheek. Samples were then stored in a small, resealable test tube and refrigerated before transfer to a -80°F daily after storefront closure.

The dust samples are useful to help understand more deeply about the participants' home environment beyond the HEH survey and what they might be exposed to on campus when attending classes. Examination of the dust samples can determine what types of microbial exposures commonly occur in students' indoor environments. Buccal swabs can be used for a variety of reasons, but were conducted as part of this study to understand how certain chemical markers like cytokine levels are related to mood and stress in participants.

3.2 Multiple-time measures

3.2.1 *Ecological Momentary Assessment*

Ecological Momentary Assessments (EMAs) involve brief questions about a participant's behavior and feelings that are answered in real-time while the participant is in their natural environment. EMAs were administered using the Beiwe mobile application[3] running on their smartphones at regularly scheduled times throughout each day. For both the Fall 2018 and the Spring 2019 cohorts, EMAs were drawn from four categories of questions: sleep questions, momentary context questions, momentary well-being questions, and an audio question. The full text of these questions are provided [see Additional File 2]. Briefly, the three sleep questions were designed to assess the duration and quality of sleep, momentary context questions were designed to determine what the participant was doing and who they were doing it with, and five well-being questions sampled participants' mood (sadness, loneliness, contentment, and stress) and energy level on a Likert scale. The audio question asked the participant to describe what they were doing and to include a brief segment of background noise.

Both the Fall 2018 and Spring 2019 cohorts received EMAs at five different times during the day. At 9am each morning they received the three sleep questions, four momentary context questions that were framed to assess the 15 minutes prior to receiving the EMAs, and five mood questions that were framed to assess how they feel at the moment. At 12pm, 3pm, and 6pm participants received EMAs that were similar to those from the morning, except that the sleep questions were removed and the audio question was added. Each night at 9pm they received the four momentary context questions and five mood questions that were framed to assess participant behaviors and feelings across the entire day.

3.3 Continuous measures

3.3.1 *Smartphone*

Passive monitoring data were collected using the Beiwe digital phenotyping platform, which is a freely available open-source system that includes mobile phone applications for Apple iOS and Google Android operating systems, and a backend server implemented in Python. The backend server was run using Amazon Web Services cloud based computing infrastructure. The backend includes a study administration web application for designing and conducting Beiwe studies and monitoring their progress, an API for sending study parameters to and receiving data from the mobile phone applications, a database for storing study state information, and an encrypted Amazon Simple Storage Service bucket for storing study data. The Beiwe developers maintain versions of the app in the Apple App store and Google Play store for easy deployment to study participants.

The full set of passive monitoring parameters are provided [see Additional File 3]. Due to differences in operating system security settings and device capabilities, different data sources were collected on Android and iOS devices. 195 out of the total 1584 participants were Android users. Basic device and operating system information (make, model, version), accelerometer, GPS, and power state data were available and collected on both devices. iOS specific data sources include gyroscope, magnetometer, the proximity of the device to the user, and whether the phone is connected to the internet by WiFi or cellular. Android specific data sources include a list of WiFi routers and Bluetooth devices in the phone's proximity, the time, duration, and hashed phone numbers for incoming and outgoing calls, and time, message length, and hashed phone numbers of incoming and outgoing text messages. To maintain participant privacy, WiFi and Bluetooth identifiers, and phone numbers are encoded with a hashing function. The function is unique however, so calls to the same destination and proximity to the same WiFi access points can be tracked across time.

The Beiwe mobile application was configured to store collected information locally and to upload it only when connected to the internet using WiFi. If an error was encountered during transmission, the app stores the data and retries transmission until receiving an indication that the data was successfully received by the backend. Each data source is stored in its own set of CSV files that are broken down and organized by timestamp. These files are encrypted on the phone before being transmit-

2 https://www.copanusa.com/sample-collection-transport-processing/floqswabs/
3 https://www.hsph.harvard.edu/onnela-lab/beiwe-research-platform/

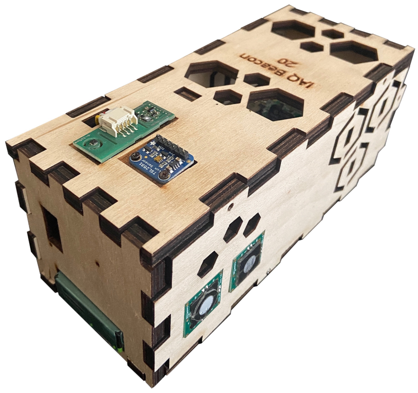

Figure 2. Building EnVironment and Occupancy (BEVO) Beacon

ted over an SSL connection to the backend. When received, the data are unencrypted, processed to correct for errors, and update received data statistics, and then re-encrypted for storage. All encryption is performed using randomly generated participant specific encryption keys.

As per the study design, participants were instructed to download and allow all permissions for the Beiwe platform. Each participant had a randomly generated identification tag that consisted of eight letters and numbers and were prompted to create their own password after entering a temporary password given to them by the study coordinator. Participants did not have direct access to their data and used the login credentials when completing the EMA surveys.

3.3.2 Wearable Activity Tracker

Participants' activity and sleep patterns were captured using the Fitbit Charge2™ wearable activity trackers. The devices require participants to input their height, weight, gender, and age to accurately calculate the number of steps taken, calories burned, and the wearer's heart rate. In addition, participants can track different exercises by selecting them from the device's interface or through the paired smartphone application. Most Fitbit products, including those supplied to the study participants, are capable of passively monitoring the wearer's sleep as long as the device detects that the user has been asleep for a minimum number of hours. The wearer's sleep is subdivided into four categories based on movement and heart rate: awake, light, deep, and rapid-eye-movement (REM). Over the past few years, many studies have looked at the accuracy and utility of using Fitbit and other personal monitoring devices in sleep studies [16, 17, 18, 19]. Results from these studies show that these devices can be useful when determining total sleep time, awake time, and the amount of time spent in REM sleep.

Similar to the swabs discussed in Section 3.1.2, each Fitbit was numbered, barcoded, and provided to participants also at the storefront location if they consented to participate in the activity monitoring component of the study. In addition to receiving the device, each participant was required to register a Fitbit account and download the smartphone application if they did not already hold an account. Participants were asked to wear the activity monitors as much as possible, removing them only when bathing, participating in aquatic activities, or charging the device. Participants were instructed to wear the Fitbit monitor over a period of at least two weeks and were free to use the device outside of the study requirements. If broken or damaged, participants were given a new device to register and use for the remaining portion of the study.

Variable	Unit	Sensor	Notes
Temperature (TF)	$^\circ F$	Adafruit SHT31-D	Occupant thermal comfort
Relative humidity (RH)	%	Adafruit SHT31-D	Occupant thermal comfort
Particulate matter (PM)	$\mu g/m^3$	Plantower PMS5003	EPA-specified criteria air pollutant with respiratory health implications
Total volatile organic compounds (TVOCs)	*ppb* (parts per billion)	Adafruit SGP30	Compounds with a wide-range of health implications from respiratory issues to cancer

Table 1. IEQ variables measured by the environmental sensors housed in the BEVO Beacon

3.3.3 Building Environment and Occupancy Beacon

The Building EnVironment and Occupancy (BEVO) beacon is a low-cost sensor platform we developed in-house that is capable of measuring multiple indoor environmental quality (IEQ) variables in addition to detecting Bluetooth and WiFi signals. The BEVO Beacon consists of a Raspberry Pi (RPi) microcomputer connected to a variety of environmental sensors arranged into a 6"×6"×4" housing made of plywood and acrylic (see Figure 2). The RPi can detect Bluetooth devices and WiFi access points in its proximity, which can help determine occupancy. The RPi is additionally capable of storing data captured by itself and the co-locating IEQ sensors on a local micro SD card, and then uploading the data to a cloud-based storage system hosted by the Texas Advanced Computing Center once connected to WiFi. Table 1 outlines the environmental sensors used in BEVO Beacon and the variables they measure. The RPi is housed in a lower compartment, separated from the sensors and wired to a small fan to help with heat regulation. To avoid the possibility of re-sampling air trapped inside the device's housing, and further help with heat management, the environmental sensors are housed in a compartment above the RPi with their inlets/exhausts exposed to the ambient air. The BEVO Beacon requires a 5B micro-USB portal connection to power.

The commercially available sensors used on the BEVO Beacon afforded us two benefits. These sensors were low-cost (<US$100), which allowed us to develop and deploy more devices than what is typically done when measuring indoor air quality. The second benefit is that these sensors serve as the base units in many other commercially available IEQ products. Using the base units rather than off-the-shelf devices ensures that there is no proprietary algorithm that alters the raw values measured by the sensors. However, reliability and accuracy are two main issues surrounding the use of low-cost sensors since they use less sophisticated electronics than high-grade reference monitors that can cost more than US$10000. Our beacon development effort is a step toward integrating low-cost sensors to achieve high home-sensing performance.

BEVO Beacons were each assigned a number and a barcode. Only the participants who completed the HEH survey (see Section 3.1.1) were eligible to receive a BEVO Beacon. Participants who were eligible and consented to participation were instructed to stop by the storefront and check out the device. At check-out, the device was supplied to the participant along with a 5V micro-USB to wall outlet adapter. Participants were instructed to power the device using any open outlet in their home. In total, we distributed 15 BEVO Beacons: five in Fall

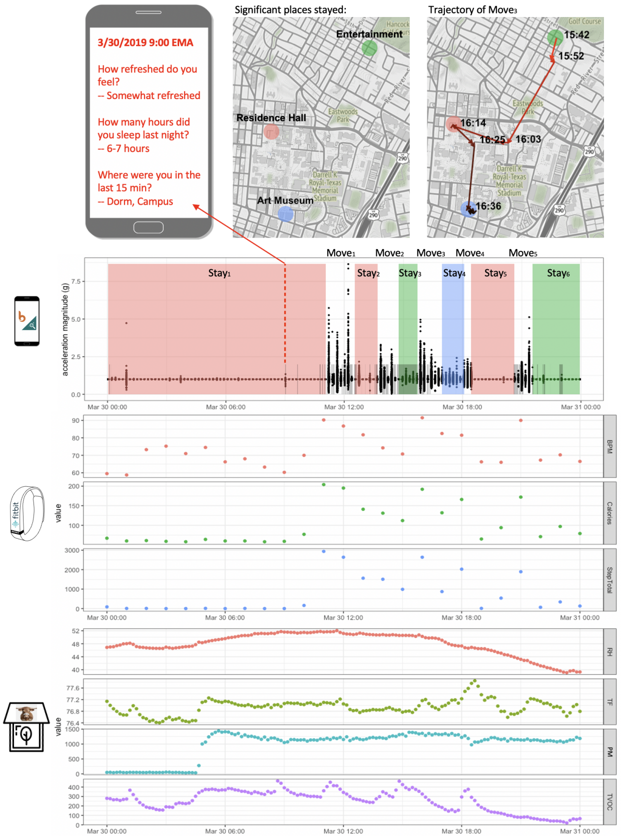

Figure 3. Data collected from the smartphone, Fitbit, and BEVO Beacon of an example participant during a given day (March 30th 2019). Plotted data modalities are: EMA (questions and answers shown against phone image background on top), GPS (clustered significant places and an example movement trajectory shown in maps on top and as vertical bands), accelerometry (black dots), screen activity (short grey bands), heart rate (BPM), calories spent in the past hour (Calories), steps taken in the past hour (StepTotal), home relative humidity (RH), home temperature in degrees Fahrenheit (TF), home particulate matter in $\mu g/m^3$ (PM), and home TVOC (TVOC).

2018 and ten in Spring 2019.

4 Data Validation

4.1 Data collected

To showcase the diverse data streams collected from our participants, we plot in Figure 3 data collected from a particular participant's smartphone, Fitbit, and BEVO Beacon on a given day (March 30th, 2019) as an example. The top third of Figure 3 shows the data types collected by the Beiwe smartphone platform. We conducted temporal clustering [20] with the raw GPS traces and processed them into periods of stay at significant places (represented by the colored vertical bands) and periods of movement between significant places (represented by the white spaces between the colored bands). We used Open Street Map API to query for the place type of the significant places found. We show the geographic location and venue type of the significant places, as well as the trajectory and time of the third transition period of the day ("Move_3") in the two maps on top. In total on March 30th, 2019, the participant made 6 stays at 3 distinct places (residence hall, art museum, and entertainment venue) and made 5 trajectories of movement between them. The participant spent her entire night and most of the morning (midnight to around 11am) at the residence hall (red band/dot), which was her main residence. Notice that even though the trajectory of Move_3 (top right map) passes through the residence hall but did not register another period of stay, suggesting that the participant merely swung by the residence hall without making a stop for an extended period of time.

During the three-week official study period, we were able to record some GPS and accelerometer data from approximately 950 participants each day. Within these days, data completeness for GPS and accelerometer followed a highly regular and well-synced daily cycle where the least percentage of participants submitted data in the early morning hours and the highest during the evening hours. Averaging the hourly completeness percentages gives us a daily average of around 65% of the participants submitting data during any given hour. In addition to mobility information, smartphone acceleration magnitude is plotted as black dots and episodes of unlocked screen as short grey bands. We observe that periods of high acceleration magnitude correspond well with periods of movement between places. Screen activity, on the other hand, varies heavily depending on the place: for example, the screen stayed unlocked during the entire Stay_3 at the entertainment venue, but locked during Stay_4 at the art museum. Moreover, the participant responded to an EMA survey at 9am, providing a self-report of her sleep quality, hours of sleep, as well as the semantics of her location at the moment. The participant did not respond to any EMA questions scheduled at other times of the day. Her answer "dorm; campus" to the location question matches with the significant place detected by GPS.

The middle third of Figure 3 shows three data streams recorded by Fitbit: heart rate (BPM), calories expense (Calories), and steps taken (StepTotal). The patterns of fluctuation of the three data streams are largely in sync with one another, with values significantly higher during the day than during the night. Three points of peak values in heart rate and calories (11am, 4pm, 9pm) visibly correspond to time intervals of high smartphone accelerometer readings, suggesting a positive correlation between smartphone accelerometry and physical activity status recorded by wearable devices. The bottom third of Figure 3 shows four data streams recorded by BEVO Beacon, all of which are metrics of indoor air quality. Note that only when the participant is located at her home location (indicated by red band in this plot, specifically $\text{Stay}_{1,2,5}$) is she exposed an environment described by these metrics; when she goes away, the metrics merely reflect her home environment status that does not affect her directly. We observe a sharp rise in humidity and PM concentration between 4-5am, which is potentially indicative of a change in the HVAC system.

4.2 Data quality

We evaluate our data's quality by two measures of completeness: first, the amount of time each participant stays in the study and continues to submit data, actively and passively; second, when the data type is continuous, the proportion of time during the entire period of participation that data are available. The first metric is important because it represents *participant compliance* and a higher value in it (or, closer to the length of the intended study period) indicates more successful participation. The latter measure is also important in that it represents *data continuity* and a higher value in it indicates fewer data-missing intervals during the total period of time a participant is submitting data.

In Figure 4 we present the completeness of four major types of data we collected, namely EMA, smartphone, Fitbit, and BEVO Beacon. In Figure 4-(A), we show the number of participants (height of bars) who submitted answers to daily EMAs for all different numbers of days (horizontal position of bars), as well as the percentage of participants (projection of red dots on vertical axis on right) who submitted answers to daily EMAs *at*

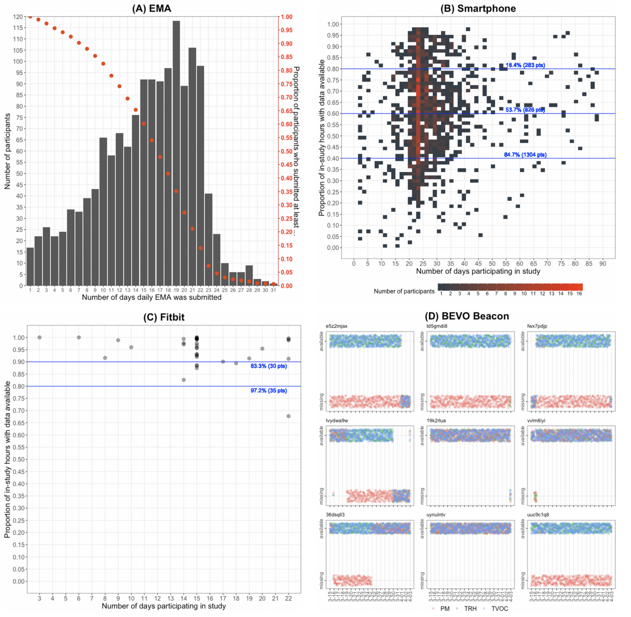

Figure 4. Completeness of four types of data collected from participants in the Fall and Spring deployments combined: (A) daily EMA (1482 participants submitted data); (B) Smartphone sensing, GPS data shown as example (1539 participants); (C) Fitbit (36 participants), and; (D) BEVO Beacon (9 participants).

least a certain number of days (horizontal position of red dots). We observe that more than 60% of participants submitted daily EMA answers for more than 14 days and more than 20% more than 21 days.

In Figure 4-(B), we show the distribution of participants with respect to the total duration of time they were contributing smartphone GPS data (i.e., compliance, on the horizontal axis) and the proportion of that duration for which their data are available (i.e., continuity, on the vertical axis). We use the color of square cells to indicate the number of participants that fall in particular compliance-continuity boundaries: the brighter-colored vertical bar between 20-25 days correspond well with our planned study length which is three weeks. Shown by the blue horizontal lines, 283 participants (18.4%) had smartphone GPS data available for more than 80% of the hours they participated in study, 53.7% of participants more than 60%, and 84.7% of participants more than 40%.

Figure 4-(C) contains the same information as (B) but plotted for Fitbit data. As opposed to aggregating the number of participants like we did for (B), because of the lower number of participants who chose to wear a Fitbit (36), we simply represented each Fitbit participant as an opaque grey dot, forming a darker cluster when more participants fall in a close region of compliance-continuity value combination. Compared to smartphone data, Fitbit data enjoyed significantly higher continuity: out of the 36 participants, 30 submitted data for more than 80% of their in-study time. This may suggest that Fitbit as a wearable device requires less human attention and interference, as opposed to smartphones which are constantly being handled and require more frequent charging, and thus are more prone to produce uninterrupted data streams.

Out of the 15 BEVO Beacons we distributed, we found that only nine recorded and uploaded data reliably and they were all from the Spring 2019 deployment. Due to such a small number of participants who returned substantial environmental sensing data, in Figure 4-(D) we simply plotted entire time series of data availability of each IEQ measure captured by BEVO Beacons (indicated by different colored dots, jittered) for each of the nine participants. Visibly, three of the nine BEVO Beacons submitted data perfectly whereas the remaining six primarily had trouble submitting PM data (red strip on the bottom). Upon inspection we found that communication errors between the sensors and the RPi accounted for a large amount of data loss. Fluctuations in power delivery between the RPi and the environmental sensors caused some environmental sensors to go offline for periods and resulted in data loss during the study.

One highly notable difference is in the number of participants who signed up for smartphone sensing compared to wearable and home environment sensing (>1000 vs. <100). We believe that both hardware availability and the incentive structure contributed to this discrepancy. First, the smartphone component of the study required the participants' own primary phone, which was widely available; whereas the Fitbit and BEVO Beacon components required extra hardware we needed to purchase, build, and provide for the participants, thus limiting the number of participants we could enroll. Second, the incentive for students to complete smartphone sensing and EMAs was to receive credits on an assignment that counted toward their final grade (with the alternative to opt out of using smartphone and only logging manually instead), which proved to be an effective strategy to improve participant compliance [21]; whereas participation in the other parts of the study was rewarded by extra experimental credits, which may not have been nearly as attractive to the students.

5 Re-use Potential

A primary advantage of our collected data is the large number of college students recruited as participants. With comprehensive momentary self-reports covering mood, type of place, social context, and activity of daily life (ADL), one can gain a representative picture of the daily experience of college students at a major university in North America. Interrelations between different aspects of mood as well as between mood and place, social context, and the concurrent ADL may be of excellent value to researchers interested in college students' well-being. Moreover, the large amounts of smartphone GPS data can serve as evidence for the temporal-geographic distributions of students' campus usage, which may be of interest to crowdsensing researchers.

Besides examining individual data types, we believe our data offer many opportunities for analyses focusing on cross-modality correlations. This can be done between two separate streams of sensing data or between sensing data and self-reported variables from momentary and pre-study surveys. A potential purpose of the between-sensors comparison is to ascertain their respective sensing capabilities of similar constructs, for example, between a smartphone's accelerometer and a Fitbit's step count and heart rate measurements. Additionally, jointly utilizing two or more sensing data streams create opportunities for novel analytical methods that help understand and predict human well-being outcomes more effectively [22]. In the case of sensor-survey comparison, a fundamental research question is whether and to what extent do variances in the sensing data correlate with variances in the self-reported data, which typically reflect the ground truth about a participant's health and behavioral states. There have been numerous digital phenotyping studies on using various mobile sensing data streams to detect mental health abnormalities and fluctuations. Our data may serve as a testbed for researchers wishing to validate different feature engineering and data mining techniques in terms of their contributions to predictive performance.

A shared characteristic of the different data types in our collection is having multiple measurements from the same participant, which may have a broader relevance to statistical analysis

in human-subject studies. Typically, when researchers collect experimental data from participants, multiple data points for each data type are collected from each participant, resulting in a situation where one has N observations from M participants and $N > M$. This is not a trivial issue because observations that belong to one participant tend to be more correlated with each other than with those from other participants, which makes the direct application of regression models onto the entirety of the N observations statistically unsound [23]. If the inter-individual variation in a particular variable is of interest and the number of participants M with data available is satisfactorily high, one appropriate approach is to aggregate the variable by the individual, such that each individual is mapped to only one value (e.g., mean value) of that variable. Interpretations made from models created on such aggregated data would be regarding the cross-participant patterns rather than within-participant ones. If one has limited amount of data available that does not justify aggregation, an appropriate solution is to use mixed effect models to account for individual differences between participants. An individual random effect could be added to both the intercept or a variable coefficient of a (generalized) linear model and the eventual selection will need to depend on model selection metrics or tests such as AIC, BIC, or a likelihood ratio test to determine whether the added effect (thus increasing variance explained) is worth the increased model complexity.

6 Conclusion

We conducted the UT1000 Project, a multi-modal data collection study using a variety of technologies and methods to monitor and understand aspects of the health, behavior, and living environments (See Figure 3) of a college student cohort of more than 1500 participants for 3 weeks. Some participants voluntarily monitored themselves for several more weeks after the official 3-week study period ended. The project is highly novel due to not only the large scale of participation but also the emphasis on incorporating the monitoring of personal living environment with health and behavior sensing to achieve a multi-faceted dashboard of human-centric information. With many types and sources of data at hand, we proposed a conceptual framework systematically organizing human-centric data modalities and their corresponding technologies and methods with respect to their temporal coverage and spatial freedom, which is further helpful for guiding data collection and research question formulation. Temporal coverage and spatial freedom overlap with ecological validity and are constrained by the unobtrusiveness of the method. Hurdles to unobtrusiveness include the size, weight, and power need of a device, requirement for human attention and maintenance, and many potential others. A general direction of evolution for human-centric design and technology is to become more portable, convenient, and user-friendly thus affording higher and higher unobtrusiveness and ecological validity for its capacity of understanding and assisting humans, until it is truly "woven into the fabric of everyday life" [24].

We were able to collect from a large participant cohort satisfactorily complete multi-modal data in terms of both data continuity and participant compliance (see Figure 4 and discussions in Section 4.2). Collection of multi-modal high-density datasets from humans in the field, like that presented in the current manuscript, has unique challenges. At enrollment, it is important to reassure potential participants that the data being collected is secure and their privacy will be respected and maintained. In the undergraduate sample utilized here, this did not appear to be a big concern for the students. Roughly 3-4% of students cited data privacy as a reason they did not want to participate. A second issue encountered was the compatibility of the smartphone application Beiwe with various software versions of iOS and Android and models of Apple and Android compatible phones. Most devices worked well, but a noticeable percentage did not. It was not always obvious what determined whether the software would work with a particular device and the study did not have the resources, nor focus for a systematic evaluation of these issues. An additional challenge was data fidelity. When collecting continuous data from multiple devices (smartphones, Fitbits, Beacons), data transfer to a database was subjected to issues of connectivity, bandwidth and participant compliance (keeping the device charged, connecting to WiFi regularly, etc.). Finally, data wrangling and clean-up is particularly challenging. The data being collected are on multiple time scales; with missing data scattered throughout, methods of data imputation and alignment create major decision points.

Several limitations exist in our study, which we would like to address in future work. First, we would like to monitor participants for a longer period of time than three weeks so that we are be able to observe more reliable patterns of behavioral variation and build more accurate personalized predictive models. Second, there is a sharp imbalance between the availability of smartphone and EMA data and the availability of Fitbit and BEVO Beacon data due to our incentive structure and hardware availability. We are preparing for a future deployment of the UT1000 Project that directly addresses these limitations by recruiting (potentially fewer) participants who will commit to longer study periods as well as increasing the number of Fitbits and BEVO Beacons distributed. Another type of data that we did not collect in the UT1000 Project is individual social media data including both the content created and the interaction patterns, which have been utilized to predict personal mental health outcomes in recent studies [25]. In our conceptual framework of human-centric data modality, social media data would fit in the medium range on both the temporal coverage and the spatial freedom axes, similar to the position of EMAs. We anticipate the collection, integration, and mining of diverse modalities of human-centric data from different technologies and methods and of various degrees of temporal coverage and spatial freedom to be key to the development of a new generation of digital solutions for personal well-being enhancement.

7 Availability of supporting data and materials

The data sets supporting the results of this article are available in the "UT1000 Data for MADS Paper" repository https://dataverse.tdl.org/dataverse/ut1000_mads. In the repository, different data types are compiled in separate datasets as follows.

- EMA https://doi.org/10.18738/T8/OPQMF3;
- Accelerometer https://doi.org/10.18738/T8/F9FHZS;
- GPS https://doi.org/10.18738/T8/1TDP7I;
- Screen unlock events https://doi.org/10.18738/T8/PAKGDB;
- Fitbit/BEVO Beacon https://doi.org/10.18738/T8/SPHD4F.

Additionally, a participant key file is provided showing our participants' gender, age, and year of participation (2018 or 2019) https://doi.org/10.18738/T8/OUUPIA.

The following additional files are also provided:

- Additional File 1, "HEH Survey Questions", a list of HEH Survey questions;
- Additional File 2, "EMA Questions", a list of EMA questions;
- Additional File 3, "Beiwe Sensing Parameters", a list of

Beiwe sensing parameters.

8 Declarations

8.1 List of abbreviations

AIC: Akaike Information Criterion; API: Application Programming Interface; BEVO: Building EnVironment and Occupancy; BIC: Bayesian Information Criterion; BPM: Beats Per Minute; CSV: Comma-Separated Values; EEG: Electroencephalography; EKG: Electrocardiography; EMA: Ecological Momentary Assessment; GPS: Global Positioning System; HEH: Home Environment and Health; HVAC: Heating, Ventilation, and Air Conditioning; IEQ: Indoor Environmental Quality; PM: Particulate Matter; REM: Rapid Eye Movement; RH: Relative Humidity; RPi: Raspberry Pi; SSL: Secure Sockets Layer; TF: Temperature in Fahrenheit; TVOC: Total Volatile Organic Compounds; USB: Universal Serial Bus.

8.2 Consent for publication

Consent for presenting de-identified individual data was obtained from participants before study started. Consent form is available upon request.

8.3 Competing Interests

The authors declare that they have no competing interests.

8.4 Funding

This work was supported by Whole Communities—Whole Health, a research grand challenge at the University of Texas at Austin, and National Science Foundation Award SES-1758835.

8.5 Author's Contributions

- CW: Conceptualization, Data curation, Formal Analysis, Methodology, Project administration, Software, Validation, Visualization, Writing – original draft, Writing – review & editing
- HF: Conceptualization, Data curation, Formal Analysis, Methodology, Resources, Software, Validation, Visualization, Writing – original draft
- JM: Conceptualization, Data curation, Methodology, Resources, Writing – original draft
- ET: Conceptualization, Data curation, Methodology, Writing – review & editing
- CJ: Conceptualization, Data curation, Methodology, Writing – review & editing
- DC: Conceptualization, Data curation, Methodology, Writing – review & editing
- KB: Conceptualization, Data curation, Methodology, Writing – review & editing
- SB: Conceptualization, Data curation, Methodology, Writing – review & editing
- GH: Conceptualization, Data curation, Funding acquisition, Methodology, Resources, Writing – review & editing
- CC: Conceptualization, Data curation, Funding acquisition, Methodology, Project administration, Resources, Supervision, Visualization, Writing – original draft, Writing – review & editing
- KK: Conceptualization, Data curation, Funding acquisition, Methodology, Project administration, Resources, Supervision, Visualization, Writing – review & editing
- SG: Conceptualization, Data curation, Funding acquisition, Methodology, Resources, Supervision, Visualization, Writing – review & editing
- DS: Conceptualization, Data curation, Funding acquisition, Methodology, Project administration, Resources, Supervision, Visualization, Writing – original draft, Writing – review & editing
- ZN: Conceptualization, Data curation, Funding acquisition, Methodology, Project administration, Resources, Supervision, Visualization, Writing – original draft, Writing – review & editing

References

1. Harari GM, Lane ND, Wang R, Crosier BS, Campbell AT, Gosling SD. Using smartphones to collect behavioral data in psychological science: Opportunities, practical considerations, and challenges. Perspectives on Psychological Science 2016;11(6):838–854.
2. Aharony N, Pan W, Ip C, Khayal I, Pentland A. Social fMRI: Investigating and shaping social mechanisms in the real world. Pervasive and Mobile Computing 2011;7(6):643–659.
3. Wang R, Chen F, Chen Z, Li T, Harari G, Tignor S, et al. StudentLife: assessing mental health, academic performance and behavioral trends of college students using smartphones. In: Proceedings of the 2014 ACM international joint conference on pervasive and ubiquitous computing ACM; 2014. p. 3–14.
4. Stopczynski A, Sekara V, Sapiezynski P, Cuttone A, Madsen MM, Larsen JE, et al. Measuring large-scale social networks with high resolution. PloS one 2014;9(4):e95978.
5. Kiukkonen N, Blom J, Dousse O, Gatica-Perez D, Laurila J. Towards rich mobile phone datasets: Lausanne data collection campaign. Proc ICPS, Berlin 2010;68.
6. Purta R, Mattingly S, Song L, Lizardo O, Hachen D, Poellabauer C, et al. Experiences measuring sleep and physical activity patterns across a large college cohort with fitbits. In: Proceedings of the 2016 ACM international symposium on wearable computers; 2016. p. 28–35.
7. Boukhechba M, Daros AR, Fua K, Chow PI, Teachman BA, Barnes LE. DemonicSalmon: Monitoring mental health and social interactions of college students using smartphones. Smart Health 2018;9:192–203.
8. Harari GM, Müller SR, Aung MS, Rentfrow PJ. Smartphone sensing methods for studying behavior in everyday life. Current opinion in behavioral sciences 2017;18:83–90.
9. Canzian L, Musolesi M. Trajectories of depression: unobtrusive monitoring of depressive states by means of smartphone mobility traces analysis. In: Proceedings of the 2015 ACM international joint conference on pervasive and ubiquitous computing; 2015. p. 1293–1304.
10. Wannenburg J, Malekian R. Physical activity recognition from smartphone accelerometer data for user context awareness sensing. IEEE Transactions on Systems, Man, and Cybernetics: Systems 2016;47(12):3142–3149.
11. Wu C, Boukhechba M, Cai L, Barnes LE, Gerber MS. Improving momentary stress measurement and prediction with bluetooth encounter networks. Smart Health 2018;9:219–231.
12. Do TMT, Blom J, Gatica-Perez D. Smartphone usage in the wild: a large-scale analysis of applications and context. In: Proceedings of the 13th international conference on multimodal interfaces; 2011. p. 353–360.
13. Cornet VP, Holden RJ. Systematic review of smartphone-based passive sensing for health and wellbeing. Journal of biomedical informatics 2018;77:120–132.

14. Webb EJ, Campbell DT, Schwartz RD, Sechrest L. Unobtrusive measures: Non-reactive research in the social sciences. Chicago: Rand McNally; 1966.
15. Barker RG, Wright HF. One boy's day: A specimen record of behavior. New York: Harper; 1951.
16. Liang Z, Martell MAC. Validity of consumer activity wristbands and wearable EEG for measuring overall sleep parameters and sleep structure in free-living conditions. Journal of Healthcare Informatics Research 2018;2(1-2):152–178.
17. de Zambotti M, Goldstone A, Claudatos S, Colrain IM, Baker FC. A validation study of Fitbit Charge 2™ compared with polysomnography in adults. Chronobiology international 2018;35(4):465–476.
18. Baron KG, Duffecy J, Berendsen MA, Mason IC, Lattie EG, Manalo NC. Feeling validated yet? A scoping review of the use of consumer-targeted wearable and mobile technology to measure and improve sleep. Sleep medicine reviews 2018;40:151–159.
19. Liang Z, Chapa-Martell MA. Accuracy of Fitbit Wristbands in Measuring Sleep Stage Transitions and the Effect of User-Specific Factors. JMIR mHealth and uHealth 2019;7(6):e13384.
20. Kang JH, Welbourne W, Stewart B, Borriello G. Extracting places from traces of locations. ACM SIGMOBILE Mobile Computing and Communications Review 2005;9(3):58–68.
21. Harari GM, Müller SR, Mishra V, Wang R, Campbell AT, Rentfrow PJ, et al. An evaluation of students' interest in and compliance with self-tracking methods: Recommendations for incentives based on three smartphone sensing studies. Social Psychological and Personality Science 2017;8(5):479–492.
22. Wu C, Barczyk AN, Craddock RC, Harari GM, Thomaz E, Shumake JD, et al. Improving prediction of real-time loneliness and companionship type using geosocial features of personal smartphone data. Smart Health 2021;20:100180.
23. Bolger N, Laurenceau JP. Intensive longitudinal methods: An introduction to diary and experience sampling research. Guilford Press; 2013.
24. Weiser M. The computer for the 21st century. ACM SIGMOBILE mobile computing and communications review 1999;3(3):3–11.
25. Mendu S, Boukhechba M, Baglione A, Baee S, Wu C, Barnes L. SocialText: A framework for understanding the relationship between digital communication patterns and mental health. In: 2019 IEEE 13th international conference on semantic computing (ICSC) IEEE; 2019. p. 428–433.

Manuscript

Click here to access/download;Manuscript;Giga_revision_manuscript_clean.

GigaScience, 2017, 1–10

doi: xx.xxxx/xxxx
Manuscript in Preparation
Paper

PAPER

Multi-Modal Data Collection for Measuring Health, Behavior, and Living Environment of Large-Scale Participant Cohorts

Congyu Wu[1,*], Hagen Fritz[2], Juan P. Maestre[2], Edison Thomaz[3], Christine Julien[3], Darla M. Castelli[4], Kaya de Barbaro[1], Sarah Kate Bearman[5], Gabriella M. Harari[7], R. Cameron Craddock[6], Kerry A. Kinney[2], Samuel D. Gosling[1,8], David M. Schnyer[1] and Zoltan Nagy[2]

[1]Department of Psychology, University of Texas at Austin and [2]Department of Civil, Environmental, and Architectural Engineering, University of Texas at Austin and [3]Department of Electrical and Computer Engineering, University of Texas at Austin and [4]Department of Kinesiology and Health Education, University of Texas at Austin and [5]Department of Educational Psychology, University of Texas at Austin and [6]Department of Diagnostic Medicine, University of Texas at Austin and [7]Department of Communication, Stanford University and [8]Melbourne School of Psychological Sciences, University of Melbourne

[*]Correspondence: congyu.wu@austin.utexas.edu

Abstract

Background As mobile technologies become ever more sensor-rich, portable, and ubiquitous, data captured by smart devices are lending rich insights into users' daily lives with unprecedented comprehensiveness and ecological validity. A number of human-subject studies have been conducted to examine the use of mobile sensing to uncover individual behavioral patterns and health outcomes, yet minimal attention has been placed on measuring living environments together with other human-centered sensing data. Moreover, the participant sample size in most existing studies falls well below a few hundred, leaving questions open about the reliability of findings on the relations between mobile sensing signals and human outcomes. **Results** To address these limitations, we developed a home environment sensor kit for continuous indoor air quality tracking and deployed it in conjunction with smartphones, Fitbits, and ecological momentary assessments in a cohort study of up to 1584 college student participants per data type for three weeks. We propose a conceptual framework that systematically organizes human-centric data modalities by their temporal coverage and spatial freedom. Then we report our study procedure, technologies and methods deployed, descriptive statistics of the collected data that reflect the participants' mood, sleep, behavior, and living environment. **Conclusions** We were able to collect from a large participant cohort satisfactorily complete multi-modal sensing and survey data in terms of both data continuity and participant compliance. Our novel data and conceptual development provide important guidance for data collection and hypothesis generation in future human-centered sensing studies.

Key words: Multi-modal sensing; human-centered computing; smartphone; Fitbit; BEVO Beacon; ecological momentary assessment; health; college students

Compiled on: March 26, 2021.
Draft manuscript prepared by the author.

1 Introduction

Human health and behavioral research is primarily conducted in laboratories under conditions that poorly approximate real-world conditions. While this model has been successful, it may miss key aspects of human behaviors that are elicited only during more natural conditions or interactions. This concern has driven interest in developing and using remote sensing technologies to measure individuals completing their normal day-to-day activities in their natural environment. An explosion in modern technologies, many of which are in common usage, now provide the ability to monitor and understand health and human behavior in ways not previously possible. Smartphones, smart home devices, wearables, and online digital behaviors provide new ways to track sleep, emotions, spatial mobility, activity, environmental exposures, and social interactions to name just a few. These technological advances offer opportunities to unobtrusively collect real-time data on a wide range of social-behavioral and health variables with less participant burden and more ecological validity than ever before [1]

In the past decade we have seen growing effort worldwide in collecting real-time sensing and experience sampling data from human participants in natural, uncontrolled settings [2, 3, 4, 5, 6, 7]. Smartphones are the staple of sensing hardware to measure different aspects of daily behavior [8]. Four main categories of behavioral patterns can be captured passively: (a) mobility trajectories, measured by GPS and further processed into location clusters that represent significant places visited [9]; (b) physical activity, measured by accelerometer and further processed into activity status labels such as walking and staying still [10]; (c) social context, reflected in different modes including phone calls, message exchange, and physical proximity detected by Bluetooth, which may be used to reconstruct social networks [11], and; (d) interaction with the device, such as screen unlock status and app usage, which are logged by the smartphone itself [12]. Additionally, ecological momentary assessment (EMA) surveys can be deployed to actively collect participant's self-reports of mood, behavior, and well-being in real time.

While many aspects of behavior and health can be captured in smartphone sensing data including EMAs with satisfactory ecological validity, we realize that other key dimensions of well-being are better measured using complementary technologies. A person spends a significant proportion of their time at home but measurements of their home environment are not generally investigated in existing studies in parallel with other aspects of daily behavior. To this end, we developed a home environment sensing device, named *BEVO Beacon*, that is capable of continuously collecting and uploading multiple measures of indoor air quality. This device can provide critical insights into a participant's living environment and evaluate its behavioral and health implications. Additionally, sleep is a critical health outcome and independent variable that is difficult to measure objectively using unobtrusive instruments. We argue it is especially beneficial to utilize the sleep measuring capability of wearable devices such as Fitbit to validate EMA answers.

Furthermore, the vast majority of existing human sensing studies used less than a few hundred participants [13]. A larger sample is needed to obtain more reliable assessments of the correlations between key behavior and health measures, especially when a large number of variables is assessed. Reflecting these considerations, we conducted a multi-modal human sensing study named *UT1000*, for which we recruited more than 1000 college students as participants over two deployments and distributed a variety of sensors and instruments including smartphone, Fitbit, BEVO Beacon, and EMA. The resulting data allow us to pursue research questions that previous data were unable to accommodate.

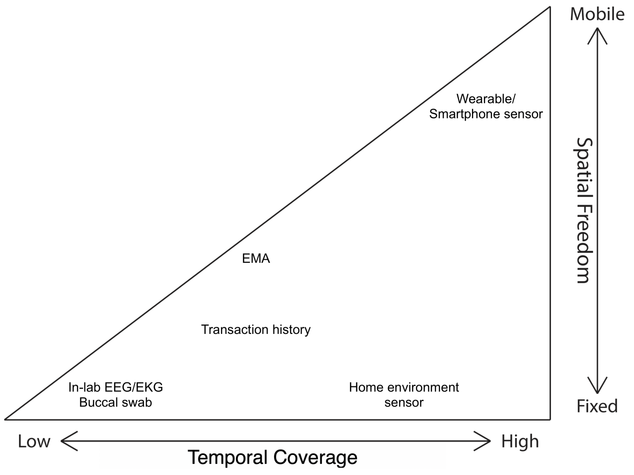

Figure 1. Human-centric Data Modality Framework

With numerous types of technologies and methods potentially available to measure individual humans' health, behavior, and environment, we begin in Section 2 by proposing a novel conceptual framework that organizes the various modalities of human-centric data based on their properties. Then, we present the study design and the types of data collected in our UT1000 Project in Section 3. We visualize the concurrent data streams collected during the study and evaluate their completeness in Section 4. In Section 5 we outline potential analytical and research applications for which our data can be useful. Through this study we are able to gain comprehensive understandings of the lives of college students and learn valuable lessons about the design, deployment, and data analysis for large-scale human sensing studies.

2 Conceptual Framework

Figure 1 illustrates the conceptual framework we devised for organizing different technologies and methods for observing human outcomes based on properties of their data collection procedure and resulting data.

A primary property of human-centric data is its *temporal coverage*, represented by the bottom horizontal axis from low (left) to high (right). Temporal coverage is defined by the inherent suitability of a data modality to monitor extended proportions of time of an individual's daily life. Data modalities that can provide only single-time observations are on the low end of the temporal coverage dimension. Examples include (1) traditional survey inventories that are designed to provide a one-time diagnosis of a potential patient and (2) medical procedures that typically require in-person clinical visits such as electroencephalography (EEG), electrocardiography (EKG), and buccal swabs. Some data collection methods or technologies accommodate measurements taken at multiple points in time, thus are placed mid-range along the temporal coverage dimension. Examples include (1) self-reports in response to EMAs delivered via mobile devices and (2) record data such as transaction history which contains user information logged at different times of user engagement with a service or services. Highest temporal coverage is achieved by continuous tracking. Various sensors embedded in devices people carry where they go and install where they stay belong in this category, such as smartphones, wearable devices, and environmental sensors used as smart home technology. Wearable devices including smartphones tend to enjoy an even higher level of temporal coverage than environmental sensors. This is because wearable devices are able to accompany and monitor the user at all

times as long as the user keeps the devices on their person and powered on, thus observing the user for longer periods of time compared to environmental sensors, which can only offer *human-centric* sensing capability when the individual in question remains within their proximity (e.g., home environment sensors are only human-centric when the person is at home).

A second property of human-centric data is its *spatial freedom*, represented by the vertical axis ranging from fixed (bottom) to mobile (top). Spatial freedom is defined by the ability of a data modality to reflect an individual's health, behavior, and environment at a variety of locations. Data modalities with greater temporal coverage tend to permit a wider range of locations where an individual can be monitored, a.k.a. higher spatial freedom. For this reason the coordinate space presented in Figure 1 is a triangle. A discrete-time measurement of low temporal coverage is typically taken at a specific location, only allowing minimal spatial freedom, thus occupying the left vertex of the triangle. However, spatial freedom may vary greatly among instruments that can track users continuously over time. For example, both a PM2.5 sensor installed at home and a smart wristband can be considered as having high temporal coverage; however, they correspond to fixed and mobile, respectively, on the spatial freedom dimension.

The temporal coverage and spatial freedom of a data modality is often governed by the unobtrusiveness [14] of the technology or method producing it. Data modalities of higher temporal coverage and spatial freedom are usually produced by devices and procedures that are more user-friendly, more portable, and overall less burdensome for the user. Unobtrusive methods allow for more naturalistic and non-interfering ways to monitor a participant's daily life, thus producing measures of greater ecological validity. We identify a major correlation between ecological validity and temporal coverage/spatial freedom: measures of high ecological validity tend to enable observations over extended periods of time and with greater mobility. However, the relation between ecological validity and temporal coverage/spatial freedom is not a necessary one. In the classic example of Barker & Wright's study of Raymond Birch [15], in which a research team followed a subject around for a whole day making observations every few minutes, temporal coverage and spatial freedom are both high however ecological validity is low, because the observation method was extremely intrusive.

The example data modalities shown in Figure 1 are based on how the generating methods are naturally and realistically carried out. For example, a buccal swab procedure takes a few minutes to complete but it requires a high level of participation and effort from the patient; therefore even though technically buccal swabs can be frequently administered, we still consider it to be a highly intrusive, one-time measurement. Many human-monitoring technologies, over their course of development, have seen themselves ascend on the temporal coverage scale and often on spatial freedom as well. For example, blood glucose testing used to require clinical visits, thus the burden was high; however, as the technology for on-body continuous glucose monitoring becomes perfected, blood glucose measurements can be obtained with unprecedentedly high unobtrusiveness, allowing its temporal coverage and spatial freedom to increase as well.

A critical challenge in interdisciplinary research joining social sciences and engineering is the high-fidelity mapping between human-centric constructs and technology-advanced methods. Our framework provides an interface between the constructs and the methods based on their temporal and spatial characteristics and implications for ecological validity and unobtrusiveness. Thus it can help social scientists locate fitting methods to measure the constructs of interest. Furthermore, this framework can be used to guide data collection and hypothesis generation concerning interrelations of different aspects of human behavior. Researchers can delineate a subarea in the triangle to serve as the scope of their own data collection. Hypotheses or research questions can be straightforwardly formulated by linking two spots on the triangle and querying the relationship between the two corresponding data modalities. Moreover, the two-dimensional space describes one individual and can be conceptually stacked up to represent a group of individuals and their data and descriptors. Linkage between slices representing different individuals can inform the generation of research questions into the relations between individual outcomes and inter-personal interactions.

3 The UT1000 Project

The UT1000 Project is a multi-modal data collection study conducted at the University of Texas at Austin to measure aspects of the health, behavior, and home environment of a large-scale participant cohort using a wide variety of technologies and methods, including traditional surveys, swabbing, EMAs, smartphone sensing, wearable trackers, and environmental sensors [1]. We undertook two deployments, one in the Fall of 2018 the other in the Spring of 2019, totaling 1584 participants (62% female) and lasting three weeks each. Participants for this study were recruited through an introductory psychology course. Enrolled students were instructed to sign up for the EMA and smartphone sensing components of the study as a class assignment that counted toward their final grade. Students who did not want to self-track using a smartphone were given the option to record their behaviors and moods by answering emailed EMA questions or keeping a daily diary. The other data modalities such as those from the wearable trackers and environmental sensors, on the other hand, were collected in return for experimental credits which the students used as partial fulfillment of the course requirements. The following subsections outline the different components of the UT1000 Project including the purpose, procedure, and types of data collected. Discussions of different study components are organized into three main categories based on the data modalities, namely *single-time*, *multiple-time*, and *continuous* measures, following an order of temporal coverage from low to high consistent with the horizontal dimension of Figure 1.

3.1 Single-time measures

3.1.1 Home Environment and Health (HEH) Survey

The Home Environment and Health (HEH) questionnaire consists of 63 questions asking students to report on home environment factors such as their current living situation including number of roommates, number and type of pets, and flooring type; their recent health and medical histories including colds, allergies, and flu shots received; and other behaviors such as hand washing frequency and use of electric scooters. A full list of the HEH questions are provided [see Additional File 1]. The purpose of this survey was to obtain a better understanding of the participants' home environment and to clarify discrepancies found in the other data streams.

The HEH questionnaire was a voluntary survey sent directly to participants via the email they provided to register for the study. Completing the HEH survey was a prerequisite for the subsequent home environment sensing component of the study discussed in Section 3.3.3. The survey was sent once during the first two weeks of the study period. Participants were asked to

1 University of Texas Institutional Review Board study number 2018-07-0035; approval date: September 25, 2018; expires: July 16, 2019.

fill out the survey based on their situation when they received it rather than some time in the past or the future. A total of 56 participants completed the HEH questionnaire, with 46 in Fall 2018 and 10 in Spring 2019.

3.1.2 *Student Environment and Buccal Swabbing*

A subset of the study participants were provided with a dust sampling kit to collect dust samples from various surfaces in their home and classroom environment. The same participants that completed the HEH survey were given the kits (N = 56). The kit consisted of six, individually-packaged Phosphate-Buffered Saline Tween-20 (PBST) wetted FLOQswabs® (manufactured by COPAN[2], Murrieta, CA) and six corresponding plastic, resealable test tubes that participants would place the swabs in after collecting samples. Participants were asked for identification in order to gather sampling materials from a refrigerator-equipped storefront created ad hoc in a convenient place at a central location of the university. Testing materials to be distributed to different participants were labeled with distinct barcodes so that we could easily trace the materials back to the participants and streamline the checkout process.

Participants followed instructions to collect samples from the interior and exterior of their front door trim, cellphone screen, living room floor, HVAC filter or air diffuser if applicable, and a desktop where they normally sit when attending university classes. After sample collection, participants sealed swabs in the provided test tubes and placed them in their refrigerator until transportation to the university lab. When participants return the testing materials, material barcodes were scanned, the identity of the participant cross-referenced to the materials, and the temperature-sensitive samples were stored until transfer to a -4°F freezer daily after storefront closure. They were asked to provide feedback on the challenges while performing home sampling and also whether they are willing to submit a buccal swab. If they consented, the research assistant in charge of operating the storefront would ask the participant to use a swab to collect a sample from the inside of their cheek. Samples were then stored in a small, resealable test tube and refrigerated before transfer to a -80°F daily after storefront closure.

The dust samples are useful to help understand more deeply about the participants' home environment beyond the HEH survey and what they might be exposed to on campus when attending classes. Examination of the dust samples can determine what types of microbial exposures commonly occur in students' indoor environments. Buccal swabs can be used for a variety of reasons, but were conducted as part of this study to understand how certain chemical markers like cytokine levels are related to mood and stress in participants.

3.2 Multiple-time measures

3.2.1 *Ecological Momentary Assessment*

Ecological Momentary Assessments (EMAs) involve brief questions about a participant's behavior and feelings that are answered in real-time while the participant is in their natural environment. EMAs were administered using the Beiwe mobile application[3] running on their smartphones at regularly scheduled times throughout each day. For both the Fall 2018 and the Spring 2019 cohorts, EMAs were drawn from four categories of questions: sleep questions, momentary context questions, momentary well-being questions, and an audio question. The full text of these questions are provided [see Additional File 2]. Briefly, the three sleep questions were designed to assess the duration and quality of sleep, momentary context questions were designed to determine what the participant was doing and who they were doing it with, and five well-being questions sampled participants' mood (sadness, loneliness, contentment, and stress) and energy level on a Likert scale. The audio question asked the participant to describe what they were doing and to include a brief segment of background noise.

Both the Fall 2018 and Spring 2019 cohorts received EMAs at five different times during the day. At 9am each morning they received the three sleep questions, four momentary context questions that were framed to assess the 15 minutes prior to receiving the EMAs, and five mood questions that were framed to assess how they feel at the moment. At 12pm, 3pm, and 6pm participants received EMAs that were similar to those from the morning, except that the sleep questions were removed and the audio question was added. Each night at 9pm they received the four momentary context questions and five mood questions that were framed to assess participant behaviors and feelings across the entire day.

3.3 Continuous measures

3.3.1 *Smartphone*

Passive monitoring data were collected using the Beiwe digital phenotyping platform, which is a freely available open-source system that includes mobile phone applications for Apple iOS and Google Android operating systems, and a backend server implemented in Python. The backend server was run using Amazon Web Services cloud based computing infrastructure. The backend includes a study administration web application for designing and conducting Beiwe studies and monitoring their progress, an API for sending study parameters to and receiving data from the mobile phone applications, a database for storing study state information, and an encrypted Amazon Simple Storage Service bucket for storing study data. The Beiwe developers maintain versions of the app in the Apple App store and Google Play store for easy deployment to study participants.

The full set of passive monitoring parameters are provided [see Additional File 3]. Due to differences in operating system security settings and device capabilities, different data sources were collected on Android and iOS devices. 195 out of the total 1584 participants were Android users. Basic device and operating system information (make, model, version), accelerometer, GPS, and power state data were available and collected on both devices. iOS specific data sources include gyroscope, magnetometer, the proximity of the device to the user, and whether the phone is connected to the internet by WiFi or cellular. Android specific data sources include a list of WiFi routers and Bluetooth devices in the phone's proximity, the time, duration, and hashed phone numbers for incoming and outgoing calls, and time, message length, and hashed phone numbers of incoming and outgoing text messages. To maintain participant privacy, WiFi and Bluetooth identifiers, and phone numbers are encoded with a hashing function. The function is unique however, so calls to the same destination and proximity to the same WiFi access points can be tracked across time.

The Beiwe mobile application was configured to store collected information locally and to upload it only when connected to the internet using WiFi. If an error was encountered during transmission, the app stores the data and retries transmission until receiving an indication that the data was successfully received by the backend. Each data source is stored in its own set of CSV files that are broken down and organized by timestamp. These files are encrypted on the phone before being transmit-

2 https://www.copanusa.com/sample-collection-transport-processing/floqswabs/
3 https://www.hsph.harvard.edu/onnela-lab/beiwe-research-platform/

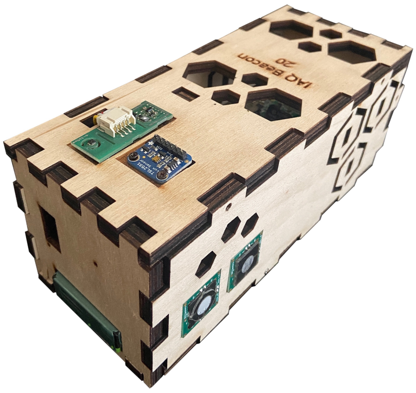

Figure 2. Building EnVironment and Occupancy (BEVO) Beacon

ted over an SSL connection to the backend. When received, the data are unencrypted, processed to correct for errors, and update received data statistics, and then re-encrypted for storage. All encryption is performed using randomly generated participant specific encryption keys.

As per the study design, participants were instructed to download and allow all permissions for the Beiwe platform. Each participant had a randomly generated identification tag that consisted of eight letters and numbers and were prompted to create their own password after entering a temporary password given to them by the study coordinator. Participants did not have direct access to their data and used the login credentials when completing the EMA surveys.

3.3.2 *Wearable Activity Tracker*

Participants' activity and sleep patterns were captured using the Fitbit Charge2^{TM} wearable activity trackers. The devices require participants to input their height, weight, gender, and age to accurately calculate the number of steps taken, calories burned, and the wearer's heart rate. In addition, participants can track different exercises by selecting them from the device's interface or through the paired smartphone application. Most Fitbit products, including those supplied to the study participants, are capable of passively monitoring the wearer's sleep as long as the device detects that the user has been asleep for a minimum number of hours. The wearer's sleep is subdivided into four categories based on movement and heart rate: awake, light, deep, and rapid-eye-movement (REM). Over the past few years, many studies have looked at the accuracy and utility of using Fitbit and other personal monitoring devices in sleep studies [16, 17, 18, 19]. Results from these studies show that these devices can be useful when determining total sleep time, awake time, and the amount of time spent in REM sleep.

Similar to the swabs discussed in Section 3.1.2, each Fitbit was numbered, barcoded, and provided to participants also at the storefront location if they consented to participate in the activity monitoring component of the study. In addition to receiving the device, each participant was required to register a Fitbit account and download the smartphone application if they did not already hold an account. Participants were asked to wear the activity monitors as much as possible, removing them only when bathing, participating in aquatic activities, or charging the device. Participants were instructed to wear the Fitbit monitor over a period of at least two weeks and were free to use the device outside of the study requirements. If broken or damaged, participants were given a new device to register and use for the remaining portion of the study.

Variable	Unit	Sensor	Notes
Temperature (TF)	$^{\circ}F$	Adafruit SHT31-D	Occupant thermal comfort
Relative humidity (RH)	%	Adafruit SHT31-D	Occupant thermal comfort
Particulate matter (PM)	$\mu g/m^3$	Plantower PMS5003	EPA-specified criteria air pollutant with respiratory health implications
Total volatile organic compounds (TVOCs)	ppb (parts per billion)	Adafruit SGP30	Compounds with a wide-range of health implications from respiratory issues to cancer

Table 1. IEQ variables measured by the environmental sensors housed in the BEVO Beacon

3.3.3 *Building Environment and Occupancy Beacon*

The Building EnVironment and Occupancy (BEVO) beacon is a low-cost sensor platform we developed in-house that is capable of measuring multiple indoor environmental quality (IEQ) variables in addition to detecting Bluetooth and WiFi signals. The BEVO Beacon consists of a Raspberry Pi (RPi) microcomputer connected to a variety of environmental sensors arranged into a 6"×6"×4" housing made of plywood and acrylic (see Figure 2). The RPi can detect Bluetooth devices and WiFi access points in its proximity, which can help determine occupancy. The RPi is additionally capable of storing data captured by itself and the co-locating IEQ sensors on a local micro SD card, and then uploading the data to a cloud-based storage system hosted by the Texas Advanced Computing Center once connected to WiFi. Table 1 outlines the environmental sensors used in BEVO Beacon and the variables they measure. The RPi is housed in a lower compartment, separated from the sensors and wired to a small fan to help with heat regulation. To avoid the possibility of re-sampling air trapped inside the device's housing, and further help with heat management, the environmental sensors are housed in a compartment above the RPi with their inlets/exhausts exposed to the ambient air. The BEVO Beacon requires a 5B micro-USB portal connection to power.

The commercially available sensors used on the BEVO Beacon afforded us two benefits. These sensors were low-cost (<US$100), which allowed us to develop and deploy more devices than what is typically done when measuring indoor air quality. The second benefit is that these sensors serve as the base units in many other commercially available IEQ products. Using the base units rather than off-the-shelf devices ensures that there is no proprietary algorithm that alters the raw values measured by the sensors. However, reliability and accuracy are two main issues surrounding the use of low-cost sensors since they use less sophisticated electronics than high-grade reference monitors that can cost more than US$10000. Our beacon development effort is a step toward integrating low-cost sensors to achieve high home-sensing performance.

BEVO Beacons were each assigned a number and a barcode. Only the participants who completed the HEH survey (see Section 3.1.1) were eligible to receive a BEVO Beacon. Participants who were eligible and consented to participation were instructed to stop by the storefront and check out the device. At check-out, the device was supplied to the participant along with a 5V micro-USB to wall outlet adapter. Participants were instructed to power the device using any open outlet in their home. In total, we distributed 15 BEVO Beacons: five in Fall

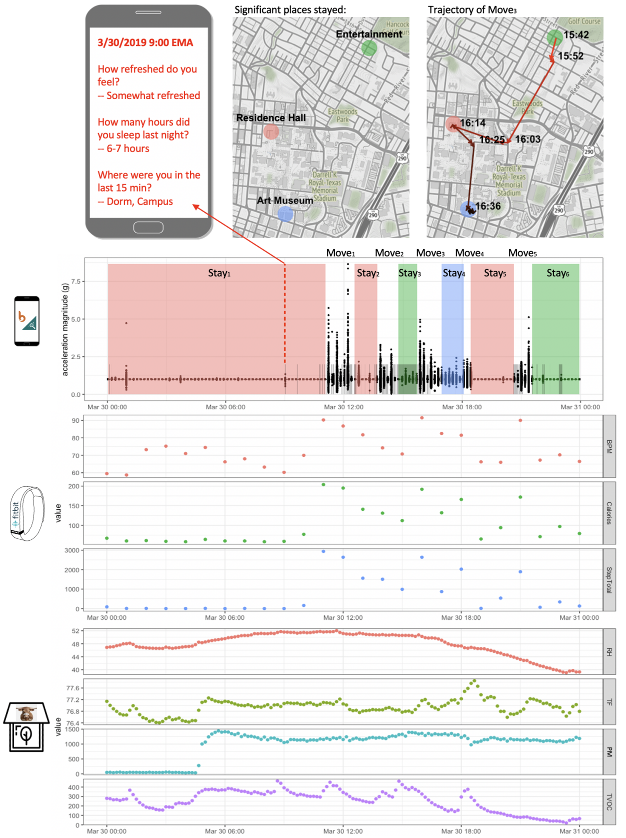

Figure 3. Data collected from the smartphone, Fitbit, and BEVO Beacon of an example participant during a given day (March 30th 2019). Plotted data modalities are: EMA (questions and answers shown against phone image background on top), GPS (clustered significant places and an example movement trajectory shown in maps on top and as vertical bands), accelerometry (black dots), screen activity (short grey bands), heart rate (BPM), calories spent in the past hour (Calories), steps taken in the past hour (StepTotal), home relative humidity (RH), home temperature in degrees Fahrenheit (TF), home particulate matter in $\mu g/m^3$ (PM), and home TVOC (TVOC).

2018 and ten in Spring 2019.

4 Data Validation

4.1 Data collected

To showcase the diverse data streams collected from our participants, we plot in Figure 3 data collected from a particular participant's smartphone, Fitbit, and BEVO Beacon on a given day (March 30th, 2019) as an example. The top third of Figure 3 shows the data types collected by the Beiwe smartphone platform. We conducted temporal clustering [20] with the raw GPS traces and processed them into periods of stay at significant places (represented by the colored vertical bands) and periods of movement between significant places (represented by the white spaces between the colored bands). We used Open Street Map API to query for the place type of the significant places found. We show the geographic location and venue type of the significant places, as well as the trajectory and time of the third transition period of the day ("Move_3") in the two maps on top. In total on March 30th, 2019, the participant made 6 stays at 3 distinct places (residence hall, art museum, and entertainment venue) and made 5 trajectories of movement between them. The participant spent her entire night and most of the morning (midnight to around 11am) at the residence hall (red band/dot), which was her main residence. Notice that even though the trajectory of Move_3 (top right map) passes through the residence hall but did not register another period of stay, suggesting that the participant merely swung by the residence hall without making a stop for an extended period of time.

During the three-week official study period, we were able to record some GPS and accelerometer data from approximately 950 participants each day. Within these days, data completeness for GPS and accelerometer followed a highly regular and well-synced daily cycle where the least percentage of participants submitted data in the early morning hours and the highest during the evening hours. Averaging the hourly completeness percentages gives us a daily average of around 65% of the participants submitting data during any given hour. In addition to mobility information, smartphone acceleration magnitude is plotted as black dots and episodes of unlocked screen as short grey bands. We observe that periods of high acceleration magnitude correspond well with periods of movement between places. Screen activity, on the other hand, varies heavily depending on the place: for example, the screen stayed unlocked during the entire Stay_3 at the entertainment venue, but locked during Stay_4 at the art museum. Moreover, the participant responded to an EMA survey at 9am, providing a self-report of her sleep quality, hours of sleep, as well as the semantics of her location at the moment. The participant did not respond to any EMA questions scheduled at other times of the day. Her answer "dorm; campus" to the location question matches with the significant place detected by GPS.

The middle third of Figure 3 shows three data streams recorded by Fitbit: heart rate (BPM), calories expense (Calories), and steps taken (StepTotal). The patterns of fluctuation of the three data streams are largely in sync with one another, with values significantly higher during the day than during the night. Three points of peak values in heart rate and calories (11am, 4pm, 9pm) visibly correspond to time intervals of high smartphone accelerometer readings, suggesting a positive correlation between smartphone accelerometry and physical activity status recorded by wearable devices. The bottom third of Figure 3 shows four data streams recorded by BEVO Beacon, all of which are metrics of indoor air quality. Note that only when the participant is located at her home location (indicated by red band in this plot, specifically $\text{Stay}_{1,2,5}$) is she exposed an environment described by these metrics; when she goes away, the metrics merely reflect her home environment status that does not affect her directly. We observe a sharp rise in humidity and PM concentration between 4-5am, which is potentially indicative of a change in the HVAC system.

4.2 Data quality

We evaluate our data's quality by two measures of completeness: first, the amount of time each participant stays in the study and continues to submit data, actively and passively; second, when the data type is continuous, the proportion of time during the entire period of participation that data are available. The first metric is important because it represents *participant compliance* and a higher value in it (or, closer to the length of the intended study period) indicates more successful participation. The latter measure is also important in that it represents *data continuity* and a higher value in it indicates fewer data-missing intervals during the total period of time a participant is submitting data.

In Figure 4 we present the completeness of four major types of data we collected, namely EMA, smartphone, Fitbit, and BEVO Beacon. In Figure 4-(A), we show the number of participants (height of bars) who submitted answers to daily EMAs for all different numbers of days (horizontal position of bars), as well as the percentage of participants (projection of red dots on vertical axis on right) who submitted answers to daily EMAs *at*

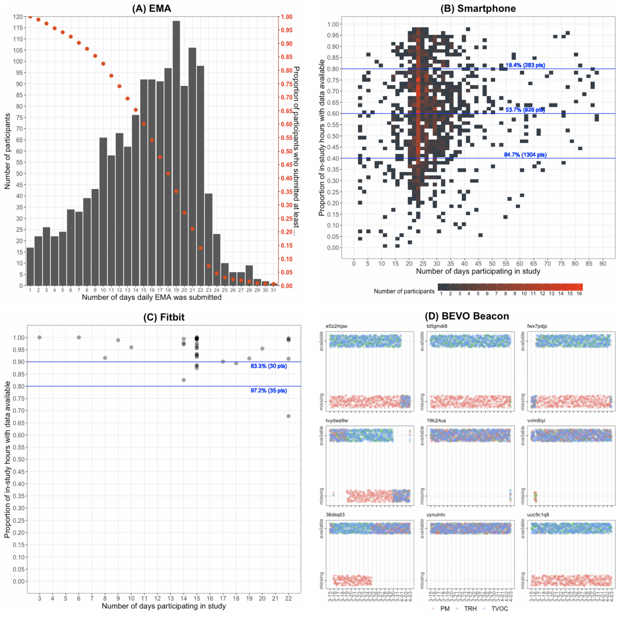

Figure 4. Completeness of four types of data collected from participants in the Fall and Spring deployments combined: (A) daily EMA (1482 participants submitted data); (B) Smartphone sensing, GPS data shown as example (1539 participants); (C) Fitbit (36 participants), and; (D) BEVO Beacon (9 participants).

least a certain number of days (horizontal position of red dots). We observe that more than 60% of participants submitted daily EMA answers for more than 14 days and more than 20% more than 21 days.

In Figure 4-(B), we show the distribution of participants with respect to the total duration of time they were contributing smartphone GPS data (i.e., compliance, on the horizontal axis) and the proportion of that duration for which their data are available (i.e., continuity, on the vertical axis). We use the color of square cells to indicate the number of participants that fall in particular compliance-continuity boundaries: the brighter-colored vertical bar between 20-25 days correspond well with our planned study length which is three weeks. Shown by the blue horizontal lines, 283 participants (18.4%) had smartphone GPS data available for more than 80% of the hours they participated in study, 53.7% of participants more than 60%, and 84.7% of participants more than 40%.

Figure 4-(C) contains the same information as (B) but plotted for Fitbit data. As opposed to aggregating the number of participants like we did for (B), because of the lower number of participants who chose to wear a Fitbit (36), we simply represented each Fitbit participant as an opaque grey dot, forming a darker cluster when more participants fall in a close region of compliance-continuity value combination. Compared to smartphone data, Fitbit data enjoyed significantly higher continuity: out of the 36 participants, 30 submitted data for more than 80% of their in-study time. This may suggest that Fitbit as a wearable device requires less human attention and interference, as opposed to smartphones which are constantly being handled and require more frequent charging, and thus are more prone to produce uninterrupted data streams.

Out of the 15 BEVO Beacons we distributed, we found that only nine recorded and uploaded data reliably and they were all from the Spring 2019 deployment. Due to such a small number of participants who returned substantial environmental sensing data, in Figure 4-(D) we simply plotted entire time series of data availability of each IEQ measure captured by BEVO Beacons (indicated by different colored dots, jittered) for each of the nine participants. Visibly, three of the nine BEVO Beacons submitted data perfectly whereas the remaining six primarily had trouble submitting PM data (red strip on the bottom). Upon inspection we found that communication errors between the sensors and the RPi accounted for a large amount of data loss. Fluctuations in power delivery between the RPi and the environmental sensors caused some environmental sensors to go offline for periods and resulted in data loss during the study.

One highly notable difference is in the number of participants who signed up for smartphone sensing compared to wearable and home environment sensing (>1000 vs. <100). We believe that both hardware availability and the incentive structure contributed to this discrepancy. First, the smartphone component of the study required the participants' own primary phone, which was widely available; whereas the Fitbit and BEVO Beacon components required extra hardware we needed to purchase, build, and provide for the participants, thus limiting the number of participants we could enroll. Second, the incentive for students to complete smartphone sensing and EMAs was to receive credits on an assignment that counted toward their final grade (with the alternative to opt out of using smartphone and only logging manually instead), which proved to be an effective strategy to improve participant compliance [21]; whereas participation in the other parts of the study was rewarded by extra experimental credits, which may not have been nearly as attractive to the students.

5 Re-use Potential

A primary advantage of our collected data is the large number of college students recruited as participants. With comprehensive momentary self-reports covering mood, type of place, social context, and activity of daily life (ADL), one can gain a representative picture of the daily experience of college students at a major university in North America. Interrelations between different aspects of mood as well as between mood and place, social context, and the concurrent ADL may be of excellent value to researchers interested in college students' well-being. Moreover, the large amounts of smartphone GPS data can serve as evidence for the temporal-geographic distributions of students' campus usage, which may be of interest to crowdsensing researchers.

Besides examining individual data types, we believe our data offer many opportunities for analyses focusing on cross-modality correlations. This can be done between two separate streams of sensing data or between sensing data and self-reported variables from momentary and pre-study surveys. A potential purpose of the between-sensors comparison is to ascertain their respective sensing capabilities of similar constructs, for example, between a smartphone's accelerometer and a Fitbit's step count and heart rate measurements. Additionally, jointly utilizing two or more sensing data streams create opportunities for novel analytical methods that help understand and predict human well-being outcomes more effectively [22]. In the case of sensor-survey comparison, a fundamental research question is whether and to what extent do variances in the sensing data correlate with variances in the self-reported data, which typically reflect the ground truth about a participant's health and behavioral states. There have been numerous digital phenotyping studies on using various mobile sensing data streams to detect mental health abnormalities and fluctuations. Our data may serve as a testbed for researchers wishing to validate different feature engineering and data mining techniques in terms of their contributions to predictive performance.

A shared characteristic of the different data types in our collection is having multiple measurements from the same participant, which may have a broader relevance to statistical analysis

in human-subject studies. Typically, when researchers collect experimental data from participants, multiple data points for each data type are collected from each participant, resulting in a situation where one has N observations from M participants and $N > M$. This is not a trivial issue because observations that belong to one participant tend to be more correlated with each other than with those from other participants, which makes the direct application of regression models onto the entirety of the N observations statistically unsound [23]. If the inter-individual variation in a particular variable is of interest and the number of participants M with data available is satisfactorily high, one appropriate approach is to aggregate the variable by the individual, such that each individual is mapped to only one value (e.g., mean value) of that variable. Interpretations made from models created on such aggregated data would be regarding the cross-participant patterns rather than within-participant ones. If one has limited amount of data available that does not justify aggregation, an appropriate solution is to use mixed effect models to account for individual differences between participants. An individual random effect could be added to both the intercept or a variable coefficient of a (generalized) linear model and the eventual selection will need to depend on model selection metrics or tests such as AIC, BIC, or a likelihood ratio test to determine whether the added effect (thus increasing variance explained) is worth the increased model complexity.

6 Conclusion

We conducted the UT1000 Project, a multi-modal data collection study using a variety of technologies and methods to monitor and understand aspects of the health, behavior, and living environments (See Figure 3) of a college student cohort of more than 1500 participants for 3 weeks. Some participants voluntarily monitored themselves for several more weeks after the official 3-week study period ended. The project is highly novel due to not only the large scale of participation but also the emphasis on incorporating the monitoring of personal living environment with health and behavior sensing to achieve a multi-faceted dashboard of human-centric information. With many types and sources of data at hand, we proposed a conceptual framework systematically organizing human-centric data modalities and their corresponding technologies and methods with respect to their temporal coverage and spatial freedom, which is further helpful for guiding data collection and research question formulation. Temporal coverage and spatial freedom overlap with ecological validity and are constrained by the unobtrusiveness of the method. Hurdles to unobtrusiveness include the size, weight, and power need of a device, requirement for human attention and maintenance, and many potential others. A general direction of evolution for human-centric design and technology is to become more portable, convenient, and user-friendly thus affording higher and higher unobtrusiveness and ecological validity for its capacity of understanding and assisting humans, until it is truly “woven into the fabric of everyday life" [24].

We were able to collect from a large participant cohort satisfactorily complete multi-modal data in terms of both data continuity and participant compliance (see Figure 4 and discussions in Section 4.2). Collection of multi-modal high-density datasets from humans in the field, like that presented in the current manuscript, has unique challenges. At enrollment, it is important to reassure potential participants that the data being collected is secure and their privacy will be respected and maintained. In the undergraduate sample utilized here, this did not appear to be a big concern for the students. Roughly 3-4% of students cited data privacy as a reason they did not want to participate. A second issue encountered was the compatibility of the smartphone application Beiwe with various software versions of iOS and Android and models of Apple and Android compatible phones. Most devices worked well, but a noticeable percentage did not. It was not always obvious what determined whether the software would work with a particular device and the study did not have the resources, nor focus for a systematic evaluation of these issues. An additional challenge was data fidelity. When collecting continuous data from multiple devices (smartphones, Fitbits, Beacons), data transfer to a database was subjected to issues of connectivity, bandwidth and participant compliance (keeping the device charged, connecting to WiFi regularly, etc.). Finally, data wrangling and clean-up is particularly challenging. The data being collected are on multiple time scales; with missing data scattered throughout, methods of data imputation and alignment create major decision points.

Several limitations exist in our study, which we would like to address in future work. First, we would like to monitor participants for a longer period of time than three weeks so that we are be able to observe more reliable patterns of behavioral variation and build more accurate personalized predictive models. Second, there is a sharp imbalance between the availability of smartphone and EMA data and the availability of Fitbit and BEVO Beacon data due to our incentive structure and hardware availability. We are preparing for a future deployment of the UT1000 Project that directly addresses these limitations by recruiting (potentially fewer) participants who will commit to longer study periods as well as increasing the number of Fitbits and BEVO Beacons distributed. Another type of data that we did not collect in the UT1000 Project is individual social media data including both the content created and the interaction patterns, which have been utilized to predict personal mental health outcomes in recent studies [25]. In our conceptual framework of human-centric data modality, social media data would fit in the medium range on both the temporal coverage and the spatial freedom axes, similar to the position of EMAs. We anticipate the collection, integration, and mining of diverse modalities of human-centric data from different technologies and methods and of various degrees of temporal coverage and spatial freedom to be key to the development of a new generation of digital solutions for personal well-being enhancement.

7 Availability of supporting data and materials

The data sets supporting the results of this article are available in the “UT1000 Data for MADS Paper" repository https://dataverse.tdl.org/dataverse/ut1000_mads. In the repository, different data types are compiled in separate datasets as follows.

- EMA https://doi.org/10.18738/T8/OPQMF3;
- Accelerometer https://doi.org/10.18738/T8/F9FHZS;
- GPS https://doi.org/10.18738/T8/1TDP7I;
- Screen unlock events https://doi.org/10.18738/T8/PAKGDB;
- Fitbit/BEVO Beacon https://doi.org/10.18738/T8/SPHD4F.

Additionally, a participant key file is provided showing our participants’ gender, age, and year of participation (2018 or 2019) https://doi.org/10.18738/T8/OUUPIA.

The following additional files are also provided:

- Additional File 1, “HEH Survey Questions", a list of HEH Survey questions;
- Additional File 2, “EMA Questions", a list of EMA questions;
- Additional File 3, “Beiwe Sensing Parameters", a list of

Beiwe sensing parameters.

8 Declarations

8.1 List of abbreviations

AIC: Akaike Information Criterion; API: Application Programming Interface; BEVO: Building EnVironment and Occupancy; BIC: Bayesian Information Criterion; BPM: Beats Per Minute; CSV: Comma-Separated Values; EEG: Electroencephalography; EKG: Electrocardiography; EMA: Ecological Momentary Assessment; GPS: Global Positioning System; HEH: Home Environment and Health; HVAC: Heating, Ventilation, and Air Conditioning; IEQ: Indoor Environmental Quality; PM: Particulate Matter; REM: Rapid Eye Movement; RH: Relative Humidity; RPi: Raspberry Pi; SSL: Secure Sockets Layer; TF: Temperature in Fahrenheit; TVOC: Total Volatile Organic Compounds; USB: Universal Serial Bus.

8.2 Consent for publication

Consent for presenting de-identified individual data was obtained from participants before study started. Consent form is available upon request.

8.3 Competing Interests

The authors declare that they have no competing interests.

8.4 Funding

This work was supported by Whole Communities—Whole Health, a research grand challenge at the University of Texas at Austin, and National Science Foundation Award SES-1758835.

8.5 Author's Contributions

- CW: Conceptualization, Data curation, Formal Analysis, Methodology, Project administration, Software, Validation, Visualization, Writing – original draft, Writing – review & editing
- HF: Conceptualization, Data curation, Formal Analysis, Methodology, Resources, Software, Validation, Visualization, Writing – original draft
- JM: Conceptualization, Data curation, Methodology, Resources, Writing – original draft
- ET: Conceptualization, Data curation, Methodology, Writing – review & editing
- CJ: Conceptualization, Data curation, Methodology, Writing – review & editing
- DC: Conceptualization, Data curation, Methodology, Writing – review & editing
- KB: Conceptualization, Data curation, Methodology, Writing – review & editing
- SB: Conceptualization, Data curation, Methodology, Writing – review & editing
- GH: Conceptualization, Data curation, Funding acquisition, Methodology, Resources, Writing – review & editing
- CC: Conceptualization, Data curation, Funding acquisition, Methodology, Project administration, Resources, Supervision, Visualization, Writing – original draft, Writing – review & editing
- KK: Conceptualization, Data curation, Funding acquisition, Methodology, Project administration, Resources, Supervision, Visualization, Writing – review & editing
- SG: Conceptualization, Data curation, Funding acquisition, Methodology, Resources, Supervision, Visualization, Writing – review & editing
- DS: Conceptualization, Data curation, Funding acquisition, Methodology, Project administration, Resources, Supervision, Visualization, Writing – original draft, Writing – review & editing
- ZN: Conceptualization, Data curation, Funding acquisition, Methodology, Project administration, Resources, Supervision, Visualization, Writing – original draft, Writing – review & editing

References

1. Harari GM, Lane ND, Wang R, Crosier BS, Campbell AT, Gosling SD. Using smartphones to collect behavioral data in psychological science: Opportunities, practical considerations, and challenges. Perspectives on Psychological Science 2016;11(6):838–854.
2. Aharony N, Pan W, Ip C, Khayal I, Pentland A. Social fMRI: Investigating and shaping social mechanisms in the real world. Pervasive and Mobile Computing 2011;7(6):643–659.
3. Wang R, Chen F, Chen Z, Li T, Harari G, Tignor S, et al. StudentLife: assessing mental health, academic performance and behavioral trends of college students using smartphones. In: Proceedings of the 2014 ACM international joint conference on pervasive and ubiquitous computing ACM; 2014. p. 3–14.
4. Stopczynski A, Sekara V, Sapiezynski P, Cuttone A, Madsen MM, Larsen JE, et al. Measuring large-scale social networks with high resolution. PloS one 2014;9(4):e95978.
5. Kiukkonen N, Blom J, Dousse O, Gatica-Perez D, Laurila J. Towards rich mobile phone datasets: Lausanne data collection campaign. Proc ICPS, Berlin 2010;68.
6. Purta R, Mattingly S, Song L, Lizardo O, Hachen D, Poellabauer C, et al. Experiences measuring sleep and physical activity patterns across a large college cohort with fitbits. In: Proceedings of the 2016 ACM international symposium on wearable computers; 2016. p. 28–35.
7. Boukhechba M, Daros AR, Fua K, Chow PI, Teachman BA, Barnes LE. DemonicSalmon: Monitoring mental health and social interactions of college students using smartphones. Smart Health 2018;9:192–203.
8. Harari GM, Müller SR, Aung MS, Rentfrow PJ. Smartphone sensing methods for studying behavior in everyday life. Current opinion in behavioral sciences 2017;18:83–90.
9. Canzian L, Musolesi M. Trajectories of depression: unobtrusive monitoring of depressive states by means of smartphone mobility traces analysis. In: Proceedings of the 2015 ACM international joint conference on pervasive and ubiquitous computing; 2015. p. 1293–1304.
10. Wannenburg J, Malekian R. Physical activity recognition from smartphone accelerometer data for user context awareness sensing. IEEE Transactions on Systems, Man, and Cybernetics: Systems 2016;47(12):3142–3149.
11. Wu C, Boukhechba M, Cai L, Barnes LE, Gerber MS. Improving momentary stress measurement and prediction with bluetooth encounter networks. Smart Health 2018;9:219–231.
12. Do TMT, Blom J, Gatica-Perez D. Smartphone usage in the wild: a large-scale analysis of applications and context. In: Proceedings of the 13th international conference on multimodal interfaces; 2011. p. 353–360.
13. Cornet VP, Holden RJ. Systematic review of smartphone-based passive sensing for health and wellbeing. Journal of biomedical informatics 2018;77:120–132.

14. Webb EJ, Campbell DT, Schwartz RD, Sechrest L. Unobtrusive measures: Non-reactive research in the social sciences. Chicago: Rand McNally; 1966.
15. Barker RG, Wright HF. One boy's day: A specimen record of behavior. New York: Harper; 1951.
16. Liang Z, Martell MAC. Validity of consumer activity wristbands and wearable EEG for measuring overall sleep parameters and sleep structure in free-living conditions. Journal of Healthcare Informatics Research 2018;2(1-2):152–178.
17. de Zambotti M, Goldstone A, Claudatos S, Colrain IM, Baker FC. A validation study of Fitbit Charge 2™ compared with polysomnography in adults. Chronobiology international 2018;35(4):465–476.
18. Baron KG, Duffecy J, Berendsen MA, Mason IC, Lattie EG, Manalo NC. Feeling validated yet? A scoping review of the use of consumer-targeted wearable and mobile technology to measure and improve sleep. Sleep medicine reviews 2018;40:151–159.
19. Liang Z, Chapa-Martell MA. Accuracy of Fitbit Wristbands in Measuring Sleep Stage Transitions and the Effect of User-Specific Factors. JMIR mHealth and uHealth 2019;7(6):e13384.
20. Kang JH, Welbourne W, Stewart B, Borriello G. Extracting places from traces of locations. ACM SIGMOBILE Mobile Computing and Communications Review 2005;9(3):58–68.
21. Harari GM, Müller SR, Mishra V, Wang R, Campbell AT, Rentfrow PJ, et al. An evaluation of students' interest in and compliance with self-tracking methods: Recommendations for incentives based on three smartphone sensing studies. Social Psychological and Personality Science 2017;8(5):479–492.
22. Wu C, Barczyk AN, Craddock RC, Harari GM, Thomaz E, Shumake JD, et al. Improving prediction of real-time loneliness and companionship type using geosocial features of personal smartphone data. Smart Health 2021;20:100180.
23. Bolger N, Laurenceau JP. Intensive longitudinal methods: An introduction to diary and experience sampling research. Guilford Press; 2013.
24. Weiser M. The computer for the 21st century. ACM SIGMOBILE mobile computing and communications review 1999;3(3):3–11.
25. Mendu S, Boukhechba M, Baglione A, Baee S, Wu C, Barnes L. SocialText: A framework for understanding the relationship between digital communication patterns and mental health. In: 2019 IEEE 13th international conference on semantic computing (ICSC) IEEE; 2019. p. 428–433.

Additional File 1

Click here to access/download
Supplementary Material
Additional File 1_titled.pdf

Additional File 2

Click here to access/download
Supplementary Material
Additional File 2_titled.pdf

Click here to access/download
Supplementary Material
Additional File 3_titled.pdf

Response to reviewers

Click here to access/download

Supplementary Material

Giga_revision_cover_letter.pdf
