# Supplementary material for: Multi-modal data collection for measuring health, behavior, and living environment of large-scale participant cohorts
Source: Gigascience. 2021 Jun 21;10(6):giab044. doi: 10.1093/gigascience/giab044 (PMC8216865; doi:10.1093/gigascience/giab044)
Supplement: giab044_Supplemental_Files [file giab044_supplemental_files.zip › Additional File 1_titled.pdf]

Additional File 1: HEH Survey Questions

Question text	Variable Type	Answer Options	Example
Which best describes your current living situation:	Str	Apartment, Dormitory, Standalone House	Apartment
How many roommates?	Int	Enter Value	2
How many housemates?	Int	Enter Value	2
How many of your housemates and roommates are female?	Int	Enter Value	2
How many of your housemates and roommates are male?	Int	Enter Value	0
How many of your housemates and roommates are nonbinary?	Int	Enter Value	1
Does anyone smoke cigarettes in the house?	Str	Yes, No	No
Does anyone vape in the house?	Str	Yes, No	No
Do you have any pets?	Str	Cat, Dog, Other, N/A	Dog
How many dogs?	Int	Enter Value	0
How many cats?	Int	Enter Value	0
How many 'other' pets?	Int	Enter Value	0
What floor is your living space on (count ground floor as 0 and count up from there)?	Int	Enter Value	5
Does the door to your apartment connect to the:	Str	indoor corridor (not open to the outside), indoor corridor (open to the outside), outdoors	Housemates
Does your living space have central air conditioning and heating?	Str	Yes, No	No
If yes, do you have control over changing the air conditioning filter?	Str	Yes, No	No
If yes, how often do you change it (in months)?	Str	Enter Response	Varies
If yes, how do you choose the filter (price, quality)?	Str	Enter Response	Varies
If yes, which filter do you choose (brand and specific product)?	Str	Enter Response	Varies

If no, how often does your apartment manager change it?	Str	Enter Response	Varies
Did you receive a swab kit as a participant in the extended study?	Str	Yes, No	No
If yes, did you swab your:	Str	AC vent, AC filter	AC Vent
Do you have control over the temperature of the home?	Str	Yes, No	No
If yes, how often per week do you typically set/change it?	Int	Enter Value	3
If yes, are you the only one who uses the thermostat?	Str	Yes, No	No
If yes, what temperature is normally chosen in winter (degrees F)?	Int	Enter Value	77
If yes, what temperature is normally chosen in summer (degrees F)?	Int	Enter Value	79
Do you open your windows to ventilate your home?	Str	Yes, No	No
If yes, how many times per week?	Int	Enter Value	3
Does your living space have water damage?	Str	Yes, No	No
If yes, indicate what space has water damage (room type, surface type):	Str	Enter Response	Varies
Does your home have moldy odor when you enter the space?	Str	Yes, No	No
Has anyone commented on bad odors when entering your home?	Str	Yes, No	No
Do you use 'air cleaner' devices?	Str	Yes, No	No
Does your house have carpet?	Str	Yes, No	No
If yes, about what % of the total area is carpeted?	Int	Enter Value	50
Does your house have:	Str	hardwood, tile floors	hardwood
If yes, about what % of the total area is hardwood?	Int	Enter Value	25
If yes, about what % of the total area is tile floor?	Int	Enter Value	25
Do you cook at home?	Str	Yes, No	No
Do you turn on the kitchen exhaust fan?	Str	Yes, No	No
In the past three weeks, have you had the flu?	Str	Yes, No	No
Have you gotten your flu shot this year?	Str	Yes, No	No
If no, are you planning on getting the flu shot?	Str	Yes, No	No
In the past three weeks, have you caught a cold?	Str	Yes, No	No
In the past three weeks, have you suffered from allergies?	Str	Yes, No	No
In the past three weeks, have you suffered from a gastrointestinal illness?	Str	Yes, No	No
In the past three weeks, have you taken antibiotics?	Str	Yes, No	No
Do you suffer from asthma (doctor-diagnosed)?	Str	Yes, No	No
Did you receive a silicon band as a participant in the extended study?	Str	Yes, No	No
If yes, did you consistently wear it as indicated?	Str	Yes, No	No
Do you use perfumes, cologne, or body lotions?	Str	Yes, No	No
If yes, how often per day do you apply these products?	Int	Enter Value	2
How often do you wash your hands per day?	Int	Enter Value	10
How often do you take a shower or bathe per week?	Int	Enter Value	10
Do you use electric scooters?	Str	Yes, No	No
If yes, how many times a day?	Int	Enter Value	1
If yes, how many times per week?	Int	Enter Value	1
