# Supplementary material for: Multi-modal data collection for measuring health, behavior, and living environment of large-scale participant cohorts
Source: Gigascience. 2021 Jun 21;10(6):giab044. doi: 10.1093/gigascience/giab044 (PMC8216865; doi:10.1093/gigascience/giab044)
Supplement: giab044_Supplemental_Files [file giab044_supplemental_files.zip › Additional File 2_titled.pdf]

Additional File 2: EMA Questions

Survey Distribution	Question Type	Question Text	Answer Options	Example
Morning only	Radio Button	How many hours did you sleep LAST NIGHT?	0 hours; did not sleep; 1-2 hours; 2-3 hours; 3-4 hours; 4-5 hours; 5-6 hours; 6-7 hours; 7-8 hours; 8-9 hours; 9-10 hours; 10-11 hours; 11-12 hours; more than 12 hours	8-9 hours
Morning only	Radio Button	How restful was your sleep?	Not at all restful; Slightly restful; Somewhat restful; Very restful	Somewhat restful
Morning only	Radio Button	How refreshed did you feel after your sleep?	Not at all refreshed; Slightly refreshed; Somewhat refreshed; Very refreshed	Somewhat refreshed
All	Checkbox	please describe your behavior during the PAST FIFTEEN MINUTES... I spent MOST of my time in the following place:	Bar; Party; Cafe; Restaurant; Campus; Fraternity; Sorority House; Gym; Home (dorm; apartment); Library; Religious facility; Store / Mall; Work; Vehicle; Friend; None of the above; other	other
All	Checkbox	please describe your behavior during the PAST FIFTEEN MINUTES...I spent MOST of my time with the following people:	Classmates; students; Co-workers; Family; Friends; No one; alone; Roommates; Significant other; Strangers; Other	Friends
All	Checkbox	please describe your behavior during the PAST FIFTEEN MINUTES...I spent MOST of my time	Attending classes; meetings; Browsing the Internet; using social media; Commuting; traveling; Doing household chores; running errands; Eating; drinking; Exercising; physical activity; sports; Resting; napping; doing nothing; Studying; reading; preparing for an exam; Talking; texting; socializing; Watching TV; movies; Working at job; None of the above; Other	napping
All	Checkbox	please describe your behavior during the PAST FIFTEEN MINUTES I spent time interacting with others by:	Talking in person; Talking on the phone; Chatting on Whatsapp or other chat app; Chatting on a dating app; Emailing; Video-chatting; Interacting on Facebook; Interacting on Instagram; Interacting on Snapchat; Interacting on Twitter; Other form of social interaction; Not applicable; was not interacting with anyone	Talking in person
All	Radio Button	I am feeling CONTENT:	Not at all; A little bit; Quite a bit; Very much	Very much
All	Radio Button	I am feeling STRESSED:	Not at all; A little bit; Quite a bit; Very much	Quite a bit
All	Radio Button	I am feeling LONELY:	Not at all; A little bit; Quite a bit; Very much	A little bit
All	Radio Button	I am feeling SAD:	Not at all; A little bit; Quite a bit; Very much	Not at all
All	Radio Button	My ENERGY LEVEL is:	Low energy; Somewhat low energy; Neutral; Somewhat high energy; High energy	High energy
