# Supplementary material for: Multi-modal data collection for measuring health, behavior, and living environment of large-scale participant cohorts
Source: Gigascience. 2021 Jun 21;10(6):giab044. doi: 10.1093/gigascience/giab044 (PMC8216865; doi:10.1093/gigascience/giab044)
Supplement: giab044_Supplemental_Files [file giab044_supplemental_files.zip › Additional File 3_titled.pdf]

Additional File 3: Beiwe Sensing Parameters

A comprehensive description of the passive data the Beiwe platform collects can be found on the developer's Wiki platform: https://github.com/onnela-lab/beiwe/wiki/Passive-Data. A short description of the data collected and used in this study is summarized below.

Data Label	Short Description	Operating Systems	Variables Collected
Accelerometer	Indication of participant movement	iOS, Android	timestamp, accuracy, x, y, z
GPS	Phone's location	iOS, Android	timestamp, latitude, longitude, altitude, accuracy
Power State	Phone screen, charging, or percentage of battery	iOS, Android	timestamp, event
Bluetooth	Records hashed MAC addresses of nearby devices	Android	timestamp, hashed ID
Reachability	Phone is connected to WiFi, cellular network, in airplane mode, or no service	iOS	timestamp, event
