# Supplementary material for: Multi-modal data collection for measuring health, behavior, and living environment of large-scale participant cohorts
Source: Gigascience. 2021 Jun 21;10(6):giab044. doi: 10.1093/gigascience/giab044 (PMC8216865; doi:10.1093/gigascience/giab044)
Supplement: giab044_Supplemental_Files [file giab044_supplemental_files.zip › Additional File 4.PDF]

Additional File 4

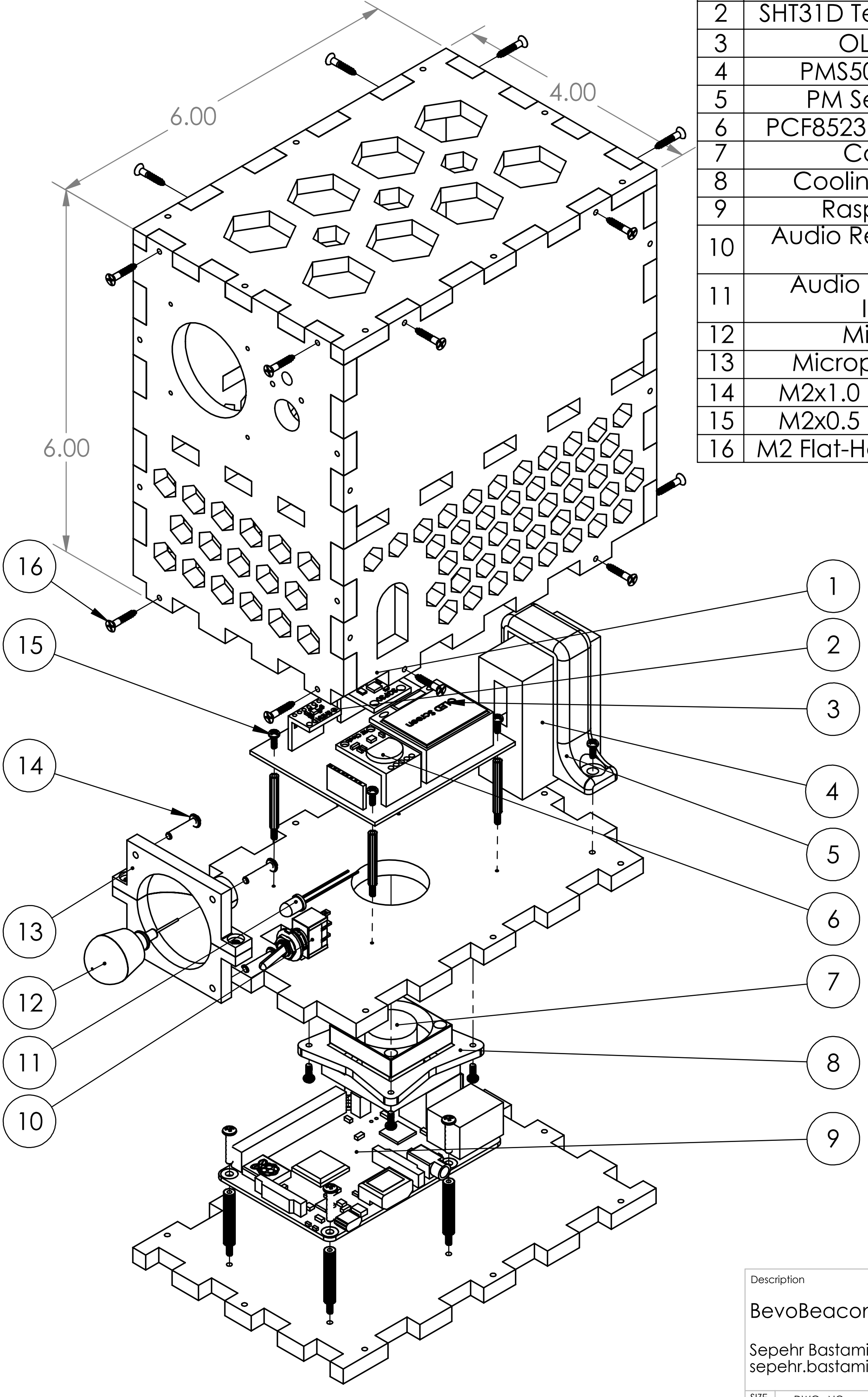

1	SGP30 Gas (TVOC) Sensor
2	SHT31D Temp. & RH Sensor
3	OLED Screen
4	PMS5003 PM Sensor
5	PM Sensor Bracket
6	PCF8523 Real-Time Clock
7	Cooling Fan
8	Cooling Fan Housing
9	RaspberryPi 3B+
10	Audio Recording Toggle Switch
11	Audio Recording LED Indicator
12	Microphone
13	Microphone Housing
14	M2x1.0 Machine Screw
15	M2x0.5 Machine Screw
16	M2 Flat-Head Wood Screw

Description

BevoBeacon 1.2

Sepehr Bastami
sepehr.bastami@utexas.edu

SIZE	DWG. NO.	REV.
A		1.8

SCALE:1:2	UNITS: IPS	SHEET 1 OF 1

SOLIDWORKS Educational Product. For Instructional Use Only.
