# Supplementary material for: Multi-modal data collection for measuring health, behavior, and living environment of large-scale participant cohorts
Source: Gigascience. 2021 Jun 21;10(6):giab044. doi: 10.1093/gigascience/giab044 (PMC8216865; doi:10.1093/gigascience/giab044)
Supplement: giab044_Response_to_Reviewer_Comments_Original_Submission [file giab044_response_to_reviewer_comments_original_submission.pdf]

Author's Response To Reviewer Comments

Close

Dear Hans,

In keeping with our recent email exchanges, we have converted our manuscript to a Data Note. We kept most of the original content and sections as they align quite well with the requirements of a Data Note. We removed some research question driven analyses and added a Re-use Potential section. We believe now the manuscript is in a good shape for a Data Note publication. We appreciate the valuable guidance you've been offering along the way.

Sincerely,
Congyu

Close
