# Supplementary material for: Multi-modal data collection for measuring health, behavior, and living environment of large-scale participant cohorts
Source: Gigascience. 2021 Jun 21;10(6):giab044. doi: 10.1093/gigascience/giab044 (PMC8216865; doi:10.1093/gigascience/giab044)
Supplement: giab044_Response_to_Reviewer_Comments_Revision_1 [file giab044_response_to_reviewer_comments_revision_1.pdf]

Author's Response To Reviewer Comments

Close

I have addressed each editorial request as specified in the last email.

Close
