# Supplementary material for: Multi-modal data collection for measuring health, behavior, and living environment of large-scale participant cohorts
Source: Gigascience. 2021 Jun 21;10(6):giab044. doi: 10.1093/gigascience/giab044 (PMC8216865; doi:10.1093/gigascience/giab044)
Supplement: giab044_Reviewer_1_Report_Original_Submission — Aku Visuri -- 1/3/2021 Reviewed [file giab044_reviewer_1_report_original_submission.pdf]

Reviewer Report

Title: Multi-Modal Data Collection for Measuring Health, Behavior, and Living Environment of Large-Scale Participant Cohorts

Version: Original Submission **Date:** 1/3/2021

Reviewer name: Aku Visuri

Reviewer Comments to Author:

This submission presents the results of a large scale deployment of environmental and behavioural sensors used to track over a thousand university students for a period of three weeks. The scale of the data collection provides significant and important results, but opposed to the authors' claims, it is not quite as unique as they claim. Numerous larger scale mobile sensing projects are ongoing in university campuses across the world. The authors, do however, provide a number of novel sensing technologies that offer further details of the participants' daily lives.

One open question that remains after reading through this submission is; what was the initial purpose of this data collection effort? What types of research questions were the authors and project managers assuming to answer? What types of research contributions was this data aimed for? And most importantly, the paper offers very little information on whether the whole data collection endeavor actually accomplished any of the goals?

Understandably, this submission could also simply be a report on how the data collection process ultimately succeeded (from the perspective of data completeness and validity, challenges, limitations, etc.) but the submission offers very little in terms of discussing these topics.

The results section reveals some interesting insights on the data but does not have any significant contributions and lacks any concrete aims or purposes for the data analysis. One could argue that there is significant proof on the validity and usability of the collected dataset and this does speak volume on the efforts of the authors, as well as proof on how well the data collection was both planned and conducted.

The discussion section of the submission is also somewhat lacking. The start of the discussion reads more as a limitations section, talking about why some aspects were left out of the overall project plan rather than focus on discussing the project outcomes. The discussion section also fails to provide any insights beyond the previously reported results and is somewhat poorly structured in terms of discussion points. There is also lack of discussion in terms of talking about this project in relation with similar projects and their findings. I would also like to hear about the authors opinions on e.g., the largest challenges of this project.

Overall, I believe this work is important and the sheer results themselves speak largely in favor of accepting this submission. The work could be significantly improved, but I would not require such improvements from the authors to simply accept their work. I am also sure that the authors do take steps elsewhere to look for more detailed research contributions of this work.

An additional minor note is that I would like to see a summary table of all the data being collected.

Level of Interest

Please indicate how interesting you found the manuscript: Choose an item.

Quality of Written English

Please indicate the quality of language in the manuscript: Choose an item.

Declaration of Competing Interests

Please complete a declaration of competing interests, considering the following questions:

- Have you in the past five years received reimbursements, fees, funding, or salary from an organisation that may in any way gain or lose financially from the publication of this manuscript, either now or in the future?
- Do you hold any stocks or shares in an organisation that may in any way gain or lose financially from the publication of this manuscript, either now or in the future?
- Do you hold or are you currently applying for any patents relating to the content of the manuscript?
- Have you received reimbursements, fees, funding, or salary from an organization that holds or has applied for patents relating to the content of the manuscript?
- Do you have any other financial competing interests?
- Do you have any non-financial competing interests in relation to this paper?

If you can answer no to all of the above, write 'I declare that I have no competing interests' below. If your reply is yes to any, please give details below.

I declare that I have no competing interests

I agree to the open peer review policy of the journal. I understand that my name will be included on my report to the authors and, if the manuscript is accepted for publication, my named report including any attachments I upload will be posted on the website along with the authors' responses. I agree for my report to be made available under an Open Access Creative Commons CC-BY license (http://creativecommons.org/licenses/by/4.0/). I understand that any comments which I do not wish to be included in my named report can be included as confidential comments to the editors, which will not be published.

Choose an item.

To further support our reviewers, we have joined with Publons, where you can gain additional credit to further highlight your hard work (see: https://publons.com/journal/530/gigascience). On publication of

this paper, your review will be automatically added to Publons, you can then choose whether or not to claim your Publons credit. I understand this statement.

Yes Choose an item.
