# Supplementary material for: Multi-modal data collection for measuring health, behavior, and living environment of large-scale participant cohorts
Source: Gigascience. 2021 Jun 21;10(6):giab044. doi: 10.1093/gigascience/giab044 (PMC8216865; doi:10.1093/gigascience/giab044)
Supplement: giab044_Reviewer_2_Report_Original_Submission — Thomas E. Doyle, Ph.D., M.E.Sc., B.E.Sc., B.Sc. -- 2/16/2021 Reviewed [file giab044_reviewer_2_report_original_submission.pdf]

Reviewer Report

Title: Multi-Modal Data Collection for Measuring Health, Behavior, and Living Environment of Large-Scale Participant Cohorts

Version: Original Submission **Date:** 2/16/2021

Reviewer name: Thomas E. Doyle, Ph.D., M.E.Sc., B.E.Sc., B.Sc.

Reviewer Comments to Author:

This is an ambitious project that I am sure will yield interesting rewults. From an high level, I appreciate that "Our novel data, conceptual development, and analytical findings provide important guidance for data collection and hypothesis generation in future human-centered sensing studies." However, it is a little disjoint without a hypothesis of the multi-modal data meaning. The paper presents sensor data for Home Environment and Health, Environment and Buccal Swabbing, Ecological Momentary Assessment, Smartphone (xyz accelerometry, GPS, bluetooth connectivity, and reachability), Wearable Activity Tracker Fitbit Charge2 (activity, sleep), BEVO (temp, relative humidity, particulate matter, total volatile organic compounds). As stated this is a diverse data set, but I believe the paper would have benefited from a demonstrable application of data fusion across sensors. I have re-read several times and it appear that the Exploratory Results subsections only report data from single sensor systems (e.g., only cell phone, only fitbit, only, etc.).

Some specific questions:

0) The abstract indicates "a cohort study of up to 1584 college student participants," but I cannot find the actual n for presented data. Please correct this.

1) Both the fitbit and smartphone collect accelerometry -- have you compared consistency or variability? Is accelerometry only used for motion detection? If not, then orientation of the smartphone must be controlled or remapped.

2) The paper notes a mix of iOS and Android devices, but this is insufficient sensor information. Can the authors comment on the specific hardware used? I have concerns about the mix and believe it impacts at least one stated result (#6 below).

3) Did the authors consider the placement or room volume of the BEVO and record the type of dwelling (basement, apartment, house, etc.). I was going to raise the saturation aspect but you have mentoined it in your results. Perhaps authors could consider broader environmental factors, such as locality to industry or other social factors. It could assist in normalizing the data.

4) Page 13 is missing a reference

5) Page 14, reducing the dimensionality by mapping to the mean assumes your data is normal distribution. Can you comment on the data distribution?

6) The authors make the claim that individual predictive algorithms perform better. It's also a potential sign of overfitting and/or variance in hardware. Please consider checking if your more general results improve when you control for the hardware.

7) The paper would be more impactful by demonstrating the fusion of sensor data. The authors describe such observations, but given that the authors have the data it should be shown in the results,

even if the results are not perfect.
Overall it is an interesting paper and I look forward to the authors applying this data and providing the data sets for others to study.

Level of Interest

Please indicate how interesting you found the manuscript: Choose an item.

Quality of Written English

Please indicate the quality of language in the manuscript: Choose an item.

Declaration of Competing Interests

Please complete a declaration of competing interests, considering the following questions:

- Have you in the past five years received reimbursements, fees, funding, or salary from an organisation that may in any way gain or lose financially from the publication of this manuscript, either now or in the future?
- Do you hold any stocks or shares in an organisation that may in any way gain or lose financially from the publication of this manuscript, either now or in the future?
- Do you hold or are you currently applying for any patents relating to the content of the manuscript?
- Have you received reimbursements, fees, funding, or salary from an organization that holds or has applied for patents relating to the content of the manuscript?
- Do you have any other financial competing interests?
- Do you have any non-financial competing interests in relation to this paper?

If you can answer no to all of the above, write 'I declare that I have no competing interests' below. If your reply is yes to any, please give details below.

I declare that I have no competing interests

I agree to the open peer review policy of the journal. I understand that my name will be included on my report to the authors and, if the manuscript is accepted for publication, my named report including any attachments I upload will be posted on the website along with the authors' responses. I agree for my report to be made available under an Open Access Creative Commons CC-BY license (http://creativecommons.org/licenses/by/4.0/). I understand that any comments which I do not wish to be included in my named report can be included as confidential comments to the editors, which will not be published.

Choose an item.

To further support our reviewers, we have joined with Publons, where you can gain additional credit to further highlight your hard work (see: https://publons.com/journal/530/gigascience). On publication of this paper, your review will be automatically added to Publons, you can then choose whether or not to claim your Publons credit. I understand this statement.

Yes Choose an item.
